# Femoral and tibial alignments in chihuahuas with patellar luxation by radiograph: Angular values and intra- and inter-observer agreement of measurements

**DOI:** 10.1371/journal.pone.0214579

**Published:** 2019-03-28

**Authors:** Masoud Aghapour, Barbara Bockstahler, Sibylle Kneissl, Alexander Tichy, Britta Vidoni

**Affiliations:** 1 Department for Companion Animals and Horses, University Clinic for Small Animals, Small Animal Surgery, University of Veterinary Medicine, Vienna, Austria; 2 Department for Companion Animals and Horses, University Clinic for Small Animals, Small Animal Surgery, Section for Physical Therapy, University of Veterinary Medicine, Vienna, Austria; 3 Department for Companion Animals and Horses, University Clinic for Small Animals, Diagnostic Imaging, University of Veterinary Medicine, Vienna, Austria; 4 Department for Biomedical Science, Platform Bioinformatics and Biostatistics, University of Veterinary Medicine, Vienna, Austria; University of Bari, ITALY

## Abstract

Measurement of fore and pelvic limb alignment in veterinary orthopedics is significant, as it is in human medicine. The establishment of reference ranges for alignments and comparing measured ranges in diseased animals to these reference values would allow veterinarians to specify the quantitative degree of an angular deformity, plan suitable treatments and evaluate treatment outcomes. Patellar luxation is a common orthopedic disease in small animal clinics. Severe grades of MPL may present with bone deformities or abnormal alignments; therefore, evaluation of the measurement methods of femoral and tibial alignment in dogs with different grades of patellar luxation to assess the accuracy and reliability of the measurements could be useful. In this retrospective study radiographs of 21 client-owned Chihuahuas that had been presented to the Small Animal Surgery of Vetmeduni Vienna from 2012–2016 with a diagnose of patellar luxation were selected. The measurements were performed on frontal, lateral and axial view radiographs to determine the femoral and tibial angles and to evaluate the intra- and inter-observer variabilities of the protocol. Radiographs of each dog were investigated by three observers. Intra-observer variability was based on measurements by each observer who repeated the protocol two times to evaluate repeatability. Inter-observer variability was based on the measurements between the three observers to evaluate the reproducibility of the protocol. The results of the study showed that 92.85% of inter-observer ICC (intra-class correlation coefficient) had high correlation, and the remaining 7.15% had good correlation. Intra-observer ICCs for measurements of the first observer were 28.57% high correlation and 50% good correlation. For the second observer, 100% high correlation was recorded, and for the third observer 71.42% high correlation and 14.28% good correlation was recorded. These results show that the selected methods have high correlation and could be used as a reliable method in veterinary orthopedics.

## Introduction

Patellar luxation is one of the most prevalent orthopedic diseases in canines, with congenital pathogenesis that can develop at a young age and may be unilateral or bilateral [1–3]. Luxation of the patella may be medial (MPL), lateral (LPL) or bidirectional. According to studies, the occurrence of MPL is significantly higher than that of LPL [3, 4] and the incidence of MPL in small breed dogs is remarkably higher than in large breeds [3].

The main underlying cause of MPL is not completely clear, but derangement of the normal anatomic structure of the hindlimb, including coxa vara, genu varum, retroversion of the femoral head and neck, distal femoral varus, superficial trochlear groove, hypoplastic medial condyles, medial torsion of the tibia, proximal tibial valgus, and medial displacement of the tibial tuberosity, have been reported as the most important predisposing factors [1,2,5].

In general, these malalignments cause alteration of the normal function of the quadriceps muscle and increase the force on the patella in the medial direction. Surgical treatment of MPL consist of bone and soft tissue reconstruction. According to the severity of the disease, grade of bone deformity, status of the patellar groove and clinical signs, different methods are used [5]. The post-operative re-luxation rate for patellar luxation is reported to be from 8% to 48%, and the re-luxation rate is more frequent in large breed dogs than in small breeds [6–8].

Bone deformities of the hind limb could be considered one of the important predispositions to MPL and therefore, evaluation of the severity of the deformity in frontal, sagittal and transverse planes could help surgeons to plan proper surgical methods. To perform deformity corrections, reference values are needed. In human medicine, using standard terminology and reference ranges of the intact skeletal structure is common, and different methods are developed, which allows physicians to differentiate normal and pathologic limb conformation [9]. As in human medicine, standard terminology and measurements have been developed in veterinary medicine [10–17].

In dogs with unilateral problems, the sound leg could be considered a reference value, but in cases of bilateral disorders, standard ranges are necessary. The normal values may vary in different breeds, and therefore evaluation of normal bone alignment in different breeds is important.

The most important methods to measure bone alignments are computed tomography and radiography. Different studies have compared these two methods in recent years [10,18]. The radiographic method is one of the most common, accessible and affordable methods in the small animal clinic. Different studies have evaluated the repeatability and reproducibility of the protocols for different breeds using radiographs [19–22].

In the present study our aims were (1) to evaluate the intra- and inter-observer variability of the protocols developed for measurement of femoral and tibial alignment in Chihuahuas with patellar luxation, (2) to report the measured values for different grades of MPL in Chihuahuas and (3) to investigate the proportion of variance of the observer and dog in this study. We hypothesized that there would be good intra- and inter-observer correlation for the values measured in this study.

## Materials and methods

In recent years different studies have evaluated hind limb alignments of different breeds. Because the occurrence of MPL in small breed dogs is higher than in large breed dogs, we investigated our database from 2012–2016 to determine the most prevalent small breed dogs that presented in Small Animal Surgery of Vetmeduni Vienna due to MPL, and the Chihuahua was selected as the most prevalent small breed dog.

Radiographs of client-owned Chihuahuas with MPL were included into this retrospective study. The age, sex, weight, and orthopedic conditions of all dogs were recorded. Dogs with concurrent orthopedic disorders related to joints other than the stifles were excluded. Radiographs of the femur had been taken in craniocaudal and axial view. The craniocaudal view was performed in tangential view with extended hip joint and parallel femur to the radiographic table. Appropriate positioning was confirmed by fluoroscopy with the lesser trochanter only partially visible, bisected fabellae by their respective femoral cortices and the vertical walls of the intercondylar notch with distinct parallel lines. The axial view radiographs of the femur were performed in dorsal recumbency with flexed hip joint such that the x-ray beam is directed down the center of the femoral diaphysis, with the cassette under the hip joint. Appropriate positioning was confirmed with fluoroscopy. The radiographs of the tibia had been taken in caudocranial and mediolateral view. In the caudocranial view, the medial aspect of the calcaneus was aligned with the intermediate tibial ridge [11,12]. As reported by Dismukes et al. the distance between the medial surface of the tuber calcaneus and intermediate tibial ridge divided by distance between two arciform grooves of the tibial cochlea and multiplied by 100. The result provided a percentage of the deviation from the center. The results larger than 50% were considered as positioning error or tibial torsion [12]. In mediolateral view, the x-ray beam was centered at the mid-diaphysis of the tibia. Distal part of the femur, entire tibia, and tarsus were covered in this projection [13]. Appropriate positioning was confirmed with superimposed femoral condyles in this view. Radiographs without superimposed femoral condyles were considered as positioning error or femur with varus or valgus deformity.

All radiographs of each dog were investigated by three observers with different levels of experience, including an expert in veterinary diagnostic imaging with 20 years’ experience, a young veterinarian with 4 years’ experience in veterinary medicine, and a small animal orthopedic surgeon with 20 years’ experience. All images were anonymized prior to the study, and the observers were trained to use the protocols before starting the measurements by using radiographs that were not included in the study; however, observers were given a written and illustrated workbook during the measurements. Measurements were performed using Cedara ProPlanner software version 3.3 (Merge Healthcare, Chicago, USA).

The intra-observer variability evaluated repeatability and was based on measurements by each observer who repeated the protocol twice with minimum of two weeks and maximum of 12 weeks between sessions. The inter-observer variability was based on measurements among the three observers and evaluated the reproducibility of the protocols.

### Measurement techniques

**Fig 1:**
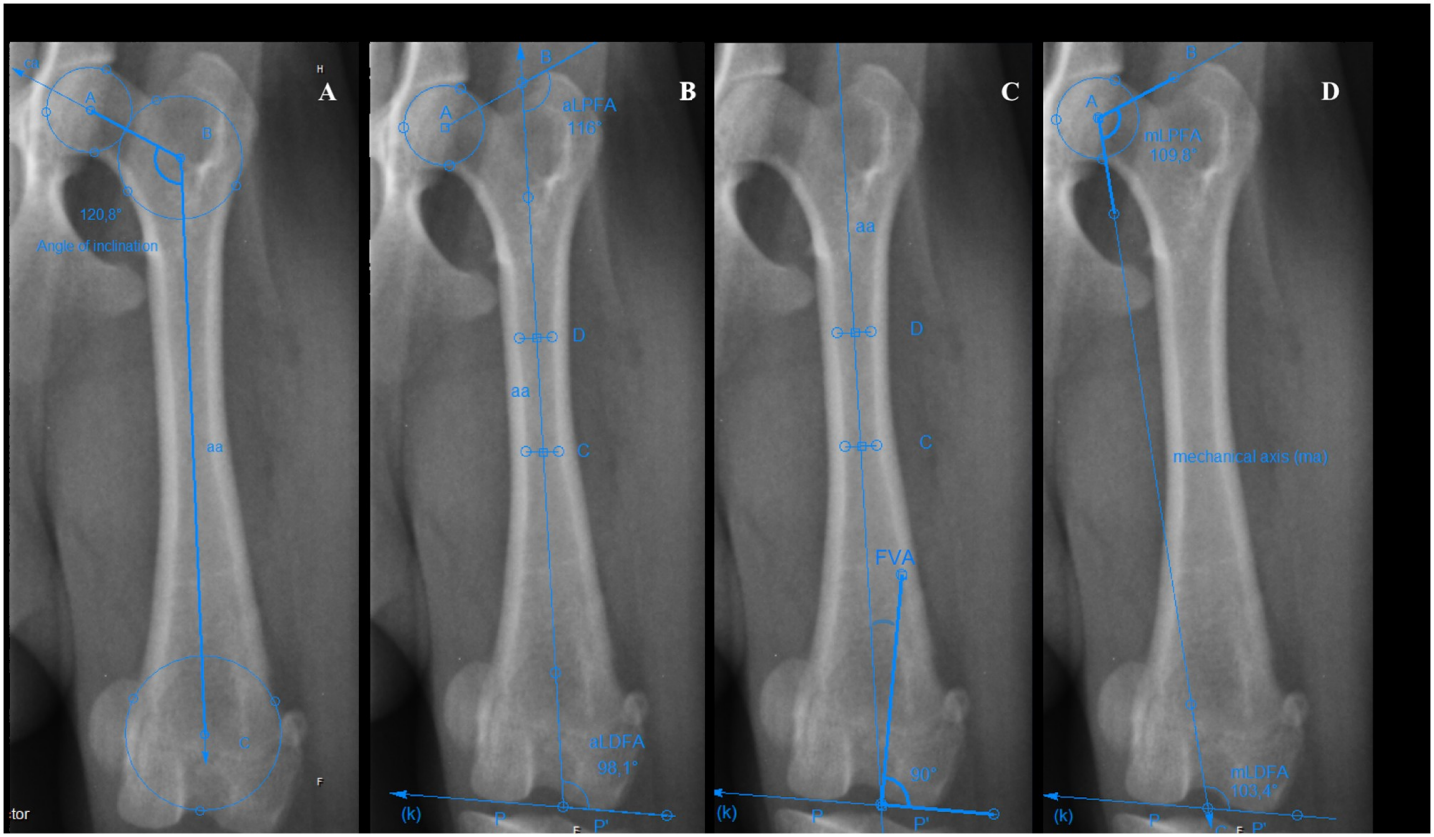
Measurements of the femoral alignment in the frontal plane. (A) Femoral neck angle or angle of inclination with the SYMAX method. Point A: center of the head of the femur; point B: center of the circle in the proximal metaphysis of the femur; point C: center of the circle in the distal metaphysis of the femur; aa: anatomic axis. (B) Anatomic lateral proximal femoral angle (aLPFA) and anatomic lateral distal femoral angle (aLDFA). Point A: center of the head of the femur; point B: proximal tip of the greater trochanter; point C: midpoint of 1/2 of the length of the femur; point D: midpoint of proximal 1/3 of the length of the femur, points P and P´: most distal convexities of the femoral condyles; K: distal joint orientation line; aa: anatomic axis. (C) Femoral varus angle (FVA). Point C: midpoint of 1/2 of the length of the femur; point D: midpoint of proximal 1/3 of the length of the femur; points P and P´: most distal convexities of the femoral condyles; K: distal joint orientation line; aa: anatomic axis. (D) Mechanical lateral proximal femoral angle (mLPFA) and mechanical lateral distal femoral angle (mLDFA). Point A: center of the head of the femur; point B: proximal tip of the greater trochanter; points P and P´: most distal convexities of the femoral condyles; K: distal joint orientation line; ma: mechanical axis.

The femoral neck angle or angle of inclination was measured on radiographs from the craniocaudal view of the femur in the sitting position with SYMAX method as described by Rumph and Hathcock (Fig 1A) [14].

**Fig 2:**
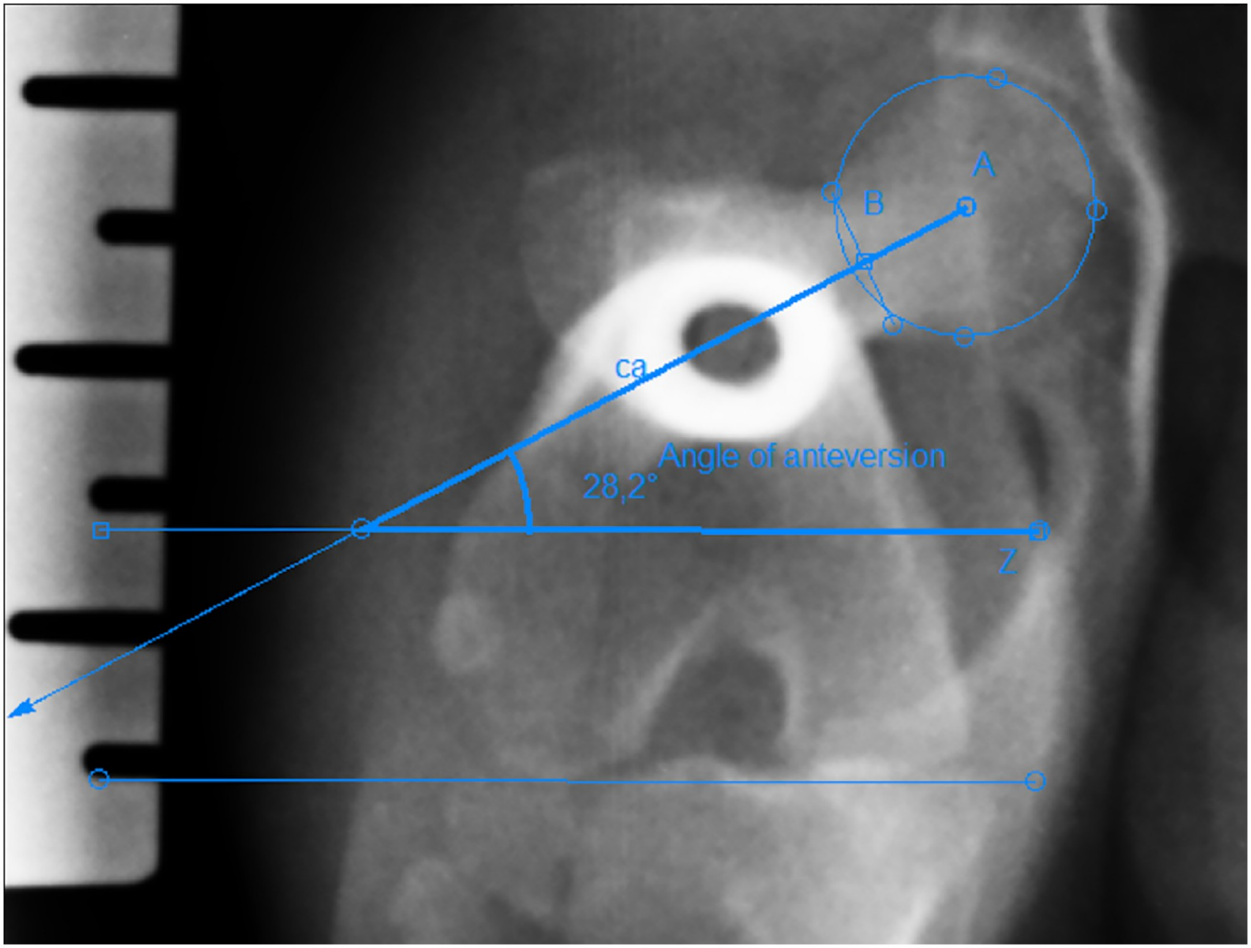
Measurement of the femoral alignments in the transverse plane. Angle of anteversion (AA). Point A: center of the head of the femur; point B: the midpoint of the femoral neck at its narrowest point between the cranial and caudal cortices; ca: cervical axis; Z: distal joint orientation line.

The anatomic lateral proximal femoral angle (aLPFA) and anatomic lateral distal femoral angle (aLDFA) were measured on radiographs from the craniocaudal view of the femur in the sitting position as described by Tomlinson et al. (Fig 1B) [9,15]. The femoral varus angle (FVA) was measured on radiographs using the craniocaudal view of the femur in the sitting position as described by Dudley et al. (Fig 1C) [10,19,23]. The mechanical lateral proximal femoral angle (mLPFA) and mechanical lateral distal femoral angle (mLDFA) were measured on radiographs using the craniocaudal view of the femur in the sitting position as described by Tomlinson et al. (Fig 1D) [9,15]. The angle of anteversion (AA) was measured on radiographs with an axially positioned femur as described by Nunamaker et al. (Fig 2) [16].

**Fig 3:**
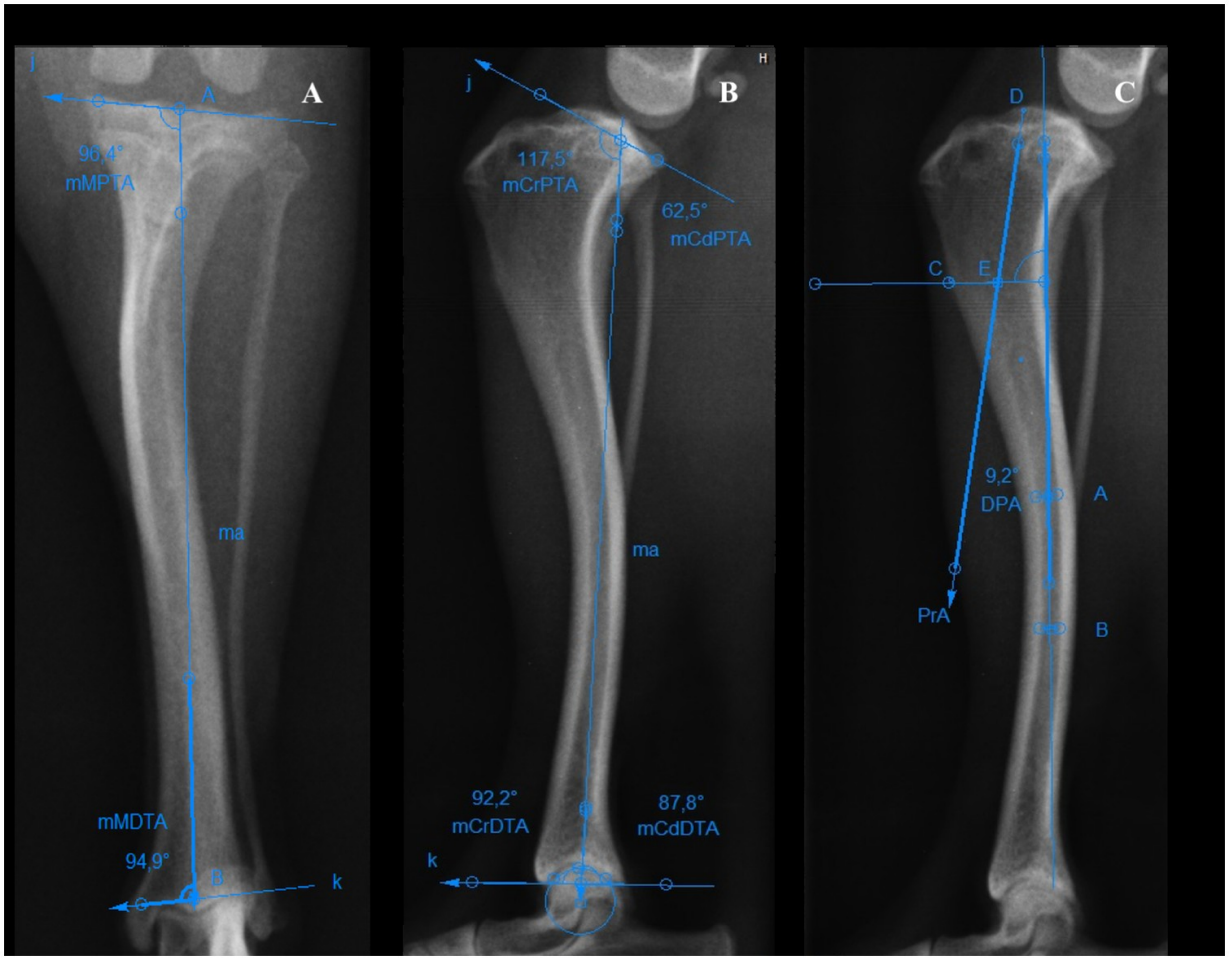
Measurements of tibial alignment in the frontal and sagittal planes. (A) Mechanical medial proximal tibial angle (mMPTA) and mechanical medial distal tibial angle (mMDTA). Point A: center of the proximal articular surface; point B: center of the distal articular surface; j: proximal joint orientation line; k: distal joint orientation line; ma: mechanical axis. (B) Mechanical cranial proximal tibial angle (mCrPTA), mechanical caudal proximal tibial angle (mCdPTA), mechanical cranial distal tibial angle (mCrDTA) and mechanical caudal distal tibial angle (mCdDTA). J: proximal joint orientation line; k: distal joint orientation line; ma: mechanical axis. (C) The distal tibial axis/proximal tibial axis angle (DPA). Point A: midpoint of the craniocaudal cortices of the tibia at 1/2 the length of the tibia; point B: midpoint of the craniocaudal cortices of the tibia at the distal 1/3 of the length of the tibia; point C: distal aspect of the tibial crest; point E: craniocaudal midpoint between the distal aspect of the tibial crest and diaphyseal tibial axis; point D: cranial aspect of the medial tibial condyle; PrA: straight line connecting the cranial aspect of the medial tibial condyle and the craniocaudal midpoint between the distal aspect of the tibial crest and the diaphyseal tibial axis.

The mechanical medial proximal tibial angle (mMPTA) and mechanical medial distal tibial angle (mMDTA) were measured on radiographs in the caudocranial view of the tibia as described by Dismukes et al. (Fig 3A) [9,12]. The mechanical cranial proximal tibial angle (mCrPTA), mechanical caudal proximal tibial angle (mCdPTA), mechanical cranial distal tibial angle (mCrDTA), mechanical caudal distal tibial angle (mCdDTA) and the distal tibial axis / proximal tibial axis angle (DPA) were measured on radiographs in the mediolateral view of the tibia as described in the literature (Fig 3B and 3C) [9,20, 24]

### Statistical analysis

Data analysis was performed using statistical software IBM SPSS statistics version 24. Measured angles were grouped as limbs with different grades of MPL from grade 1 to grade 4 according to the grading system modified from Singleton [2]. The mean and standard deviation for each angle with different grades of MPL were calculated.

The intra-class correlation coefficient (ICC) was calculated from the first and second round of measurements by each observer (intra-observer) and repeated measurements by all observers (inter-observer) for each angle. The ICC ranged from 0 (no agreement) to 1 (perfect agreement). An ICC > 0.75 was considered high correlation, 0.74 > ICC > 0.60 was considered good correlation, 0.59 > ICC > 0.4 was considered fair correlation and an ICC less than 0.40 was considered poor correlation [25].

The measurements for each observer were grouped as the first and second round of measurements to evaluate the intra-observer variability, and the measurements of all 3 observers were grouped to evaluate the inter-observer variabilities.

The group range is the absolute value of the difference between the first and second measurement for each angle by each observer. It represents how the difference between sessions for the same angle with the same methods could exist, and it can be considered a significant factor to assess the human error in radiographic measurements. In this study, 4 degrees of difference were considered negligible because this amount of difference may not affect the surgical intervention or surgical outcomes. The difference between the first and second round of measurements by each observer for each angle was calculated, and the intra-observer group ranges were recorded. The differences between the first and second round of measurements for all observers were calculated, and the inter-observer group ranges were recorded. The group ranges were categorized as 0–2°, 2.1–4°, 4.1–6°, 6.1–8°, 8.1–10° and greater than 12°. The group ranges are shown in S1 File.

Measurements for each angle were analyzed using a mixed effect linear model with the extremity and observer as random effects and measurement repetition as the fixed effect.

## Results

Hind limb radiographs of 21 (42 limbs) client-owned Chihuahuas were investigated in this study. Six of the evaluated dogs were male (2 intact, 4 neutered), and 15 dogs were female (9 intact, 6 spayed). The mean ± SD (range) age of the dogs was 2.91 ± 2.63 years (0.58–9.33 years). The mean ± SD (range) body weight was 3.06 ± 1.05 kg (1.6–5.7 kg).

Evaluated Chihuahuas had different directions of patellar luxation, including medial patellar luxation (Grade 1: 3 limbs, Grade 2: 6 limbs, Grade 3: 28 limbs, Grade 4: 5 limbs) and lateral patellar luxation (Grade 1: 3 limbs, Grade 2: 1 limb).

Measured angles from all observers were grouped as limbs with different grades of MPL from grade 1 to grade 4 [2]. The mean and standard deviation for each angle with different grades of MPL were calculated. The mean ± SD for the ICA, aLPFA, aLDFA, FVA, mLPFA, mLDFA, AA, mMPTA, mMDTA, mCrPTA, mCdPTA, mCrDTA, mCdDTA and DPA are shown in Table 1.

**Table 1:** Hind limb alignments (mean ± SD) of Chihuahuas with different grades of MPL in frontal, transverse and sagittal planes using radiographic methods.

Angle	Grade 1	Grade 2	Grade3	Grade4
	Mean ± SD	Mean ± SD	Mean ± SD	Mean ± SD
**ICA (°)**	123.65 ± 3.15	122.89 ± 2.72	123.72 ± 8.96	121.76 ± 4.94
**aLPFA (°)**	122.01 ± 4.12	117.15 ± 6.01	115.41 ± 7.17	111.96 ± 4.05
**aLDFA (°)**	99.71 ± 2.25	101.55 ± 3.76	100.23 ± 4.52	111.92 ± 9.87
**FVA (°)**	9.28 ± 2.49	11.28 ± 3.77	10.12 ± 4.46	21.21 ± 9.25
**mLPFA (°)**	116.9 ± 6.03	113.41 ± 6.43	111.52 ± 6.86	110.71 ± 5.24
**mLDFA (°)**	104.01 ± 3.14	105.18 ± 1.49	104.2 ± 3.12	110.7 ± 7.95
**AA (°)**	24.86 ± 5.94	21.66 ± 3.9	22.69 ± 6.03	19.08 ± 10.17
**mMPTA (°)**	97.11 ± 2.65	97.15 ± 3.94	96.85 ± 2.38	100.18 ± 3.83
**mMDTA (°)**	96.23 ± 2.76	95.53 ± 2.85	94.64 ± 2.96	95.81 ± 1.67
**mCrPTA (°)**	123.29 ± 6.30	121.37 ± 5.68	119.26 ± 5.46	122.3 ± 9.84
**mCdPTA (°)**	56.68 ± 6.49	58.56 ± 5.7	60.79 ± 5.87	57.69 ± 10.06
**mCrDTA (°)**	90.41 ± 3.31	90.58 ± 4.44	90.25 ± 3.9	94.36 ± 6.32
**mCdDTA(°)**	89.37 ± 2.72	89.0 ± 4.72	89.73 ± 3.8	85.5 ± 6.44
**DPA (°)**	11.88 ± 5.25	10.85 ± 5.76	8.85 ± 4.92	9.31 ± 5.27

ICA: angle of inclination, aLPFA: anatomic lateral proximal femoral angle, aLDFA: anatomic lateral distal femoral angle, FVA: femoral varus angle, mLPFA: mechanical lateral proximal femoral angle, mLDFA: mechanical lateral distal femoral angle, AA: angle of anteversion, mMPTA: mechanical medial proximal tibial angle, mMDTA: mechanical medial distal tibial angle, mCrPTA: mechanical cranial proximal tibial angle, mCdPTA: mechanical caudal proximal tibial angle, mCrDTA: mechanical cranial distal tibial angle, mCdDTA: mechanical caudal distal tibial angle, DPA: distal tibial axis/proximal tibial axis angle.

The intra-observer ICC for measurements of the first observer was greater than 0.75 for 28.57% of the measurements, indicating high correlation, and between 0.6 and 0.74 for 50% of the measurements, indicating good correlation. For the second observer, all of the measurements (100%) were greater than 0.75, indicating high correlation. For the third observer, 71.42% of the measurements were greater than 0.75, indicating high correlation, and just 14.28% of the measurements were between 0.6 and 0.74, indicating good correlation.

The inter-observer ICC was greater than 0.75 for 92.85% of the measurements, indicating high correlation, and the other 7.15% of the measurements were between 0.6 and 0.74, indicating good correlation. The intra- and inter-observer ICC are shown in Table 2.

**Table 2:** Intra-class correlation coefficient (ICC) for inter- and intra-observer variability between and within the observers.

Angles	Inter-observer	Intra-observer		
		Observer 1	Observer 2	Observer 3
**ICA**	0.96	0.81	0.95	0.94
**aLPFA**	0.96	0.80	0.98	0.95
**aLDFA**	0.95	0.74	0.99	0.98
**FVA**	0.94	0.73	0.95	0.97
**mLPFA**	0.95	0.69	0.98	0.94
**mLDFA**	0.95	0.80	0.99	0.94
**AA**	0.86	0.66	0.97	0.81
**mMPTA**	0.97	0.89	0.95	0.90
**mMDTA**	0.86	0.63	0.85	0.48
**mCrPTA**	0.83	0.66	0.94	0.88
**mCdPTA**	0.81	0.63	0.95	0.90
**mCrDTA**	0.72	0^a^	0.95	0.72
**mCdDTA**	0.76	0^a^	0.95	0.70
**DPA**	0.75	0.10	0.94	0.54

^a^The values were set to zero due to the negative covariance.

The percentage of the intra-observer group ranges that were less than 4 degrees included 67.08% (femur: 70.52%, tibia: 63.65%) for the first observer, 94.26% (femur: 93.92%, tibia: 94.6%) for the second observer and 80.86% (femur: 88.38%, tibia: 73.34%) for the third observer. Furthermore, 73.37% (femur: 77.54%, tibia: 69.2%) of the inter-observer group ranges were less than 4 degrees. The percentages of the intra- and inter-observer group ranges are shown in Table 3.

**Table 3:** The percentages of the intra- and inter-observer group ranges* that were less than 4 degrees.

Angles		Inter-observer	Intra-observer		
			Observer 1	Observer 2	Observer 3
**ICA**		92.3%	71.79%	90%	92.85%
**aLPFA**		73.17%	63.15%	80%	87.80%
**aLDFA**		80.48%	87.37%	100%	97.56%
**FVA**		76.92%	78.94%	97.5%	97.56%
**mLPFA**		61.53%	55.26%	90%	85.36%
**mLDFA**		90%	87.17%	100%	97.56%
**AA**		68.42%	50%	100%	60%
**mMPTA**		97.36%	94.73%	97.43%	97.36%
**mMDTA**		89.47%	81.08%	92.3%	76.31%
**mCrPTA**		61.53%	51.42%	90%	76.92%
**mCdPTA**		52.63%	45.71%	95%	82.05%
**mCrDTA**		55.26%	54.54%	97.5%	61.53%
**mCdDTA**		69.23%	51.42%	95%	69.23%
**DPA**		58.98%	66.66%	95%	50%
**Sum**	**Femur**	77.54%	70.52%	93.92%	88.38%
	**Tibia**	69.2%	63.65%	94.6%	73.34%
	**All**	73.37%	67.08%	94.26%	80.86%

*The group range is the absolute value of the difference between the first and second measurement for each angle by each observer. It represents the difference between sessions for the same angle with the same methods.

The proportions of the variance in this study were significantly attributed to the extremities, except the DPA. The proportion of the variance for DPA was 44.45% for the extremities and 55.55% for the observers. The proportion of the variance for extremity and observer are shown in Table 4.

**Table 4:** Percentage of the variables in the measurement of each parameter.

Angle	Extremity %	Observer %
**ICA (SYMAX)**	99.76	3.24
**aLPFA**	99.33	0.67
**aLDFA**	99.63	0.37
**FVA**	99.55	0.45
**mLPFA**	99.28	0.72
**mLDFA**	99.7	0.3
**AA**	93.8	6.2
**mMPTA**	99.27	7.23
**mMDTA**	77.03	29.97
**mCrPTA**	99.06	0.94
**mCdPTA**	98.99	1.01
**mCrDTA**	99.9	0.1
**mCdDTA**	99.9	0.1
**DPA**	44.45	55.55

## Discussion

This study was designed to assess the reliability of described methods to measure the femoral and tibial alignment in Chihuahuas using radiographs. We hypothesized that there are good intra- and inter-observer correlations for the values measured in this study. The results show that good intra-observer correlation for observer one and high intra-observer correlations for observers two and three were recorded. The inter-observer correlation among the observers was high in this study. These results show that the radiographic method for the measurement of femoral and tibial alignment has statically good, or in most of the cases even high, intra- and inter-observer correlations.

One of the most important reasons for the difference between the observers is the level of experience. In this study three observers with different level of experience evaluated the radiographs. The observer one was an expert in veterinary diagnostic imaging with 20 years’ experience, the second observer was a young veterinarian with 4 years’ experience in veterinary medicine and the third observer was an expert in small animal orthopedics with 20 years’ experience. The results showed that the measurements of the observers two and three were more accurate than those recorded for observer one. Most of these measurement methods were new for observer two but not for the observer three. Despite the fact that the observer one was an experienced expert in diagnostic imaging, she did not perform these measurements routinely, therefore the observer one had not that much experience as observer three, who did these measurements frequently before. The observer two in this study had the lowest level of experience within the observers, but he trained the measurement before the study on several non-related radiographs; therefore, the results of his measurements had high correlation. The authors believe that the experience of the observers played important role in this study, the observers who trained or performed the measurement frequently before, had better results.

According to the results evaluation of the femoral alignment had a better correlation than evaluation of the tibia. However, measurements in the frontal plane were more accurate than those in the sagittal plane. Evaluation of the measurements of the tibial angles showed that there is a better correlation for proximal tibial alignment compared with distal tibial alignment. One of the reasons for this difference may be the landmarks. Identification of landmarks in the frontal plane is easier than in the sagittal or transverse planes, because fewer numbers of landmarks are identified in the frontal plane. The anatomy of the bone is also significant, and the different results between the femur and tibia are due to the different anatomic shape of the bones. The positioning of the dogs to perform radiographic imaging could influence the results, especially in dogs with muscle contracture or bone malformation; therefore, having a standard imaging protocol to achieve consistent outcomes should be considered.

The muscle contracture should be considered as an important factor that may affect the radiographic measurements. All of the radiographs in this study were performed under general anesthesia to minimize the errors and indicate muscle relaxation.

In different articles different ranges are considered a negligible error amount. In this study the authors agreed that 4 degrees of measurement error was a negligible error amount. We believe that up to 4 degrees of error will not influence the final results or surgical interventions. In different studies, the effect of positioning errors was assessed [19,22,26], but no investigations evaluated the influence of measurement errors on surgical outcomes. Evaluation of the influence of measurement errors on surgical outcomes shows how many degrees of error can be ignored during investigations. Further research in this area is necessary.

Previous studies have evaluated the reliability of the radiographic method. These measurements were repeatable and reproducible but not accurate [19]. The difference between the present study and previous studies is that in this study, a large number of femoral and tibial angles (14 angles) in a specified dog breed (Chihuahuas) were evaluated according to intra- and inter-observer differences. In this study, most of the angles had high or good intra-class correlation coefficients, indicating high and good repeatability and reproducibility. Only a few had fair or poor reliability. The reason for these poor results may be positioning mistakes, measurement errors and errors in the notation of the measured ranges [22]. According to the literature, radiographs are vulnerable to positioning errors [19,22], and the positioning of dogs with severe degrees of bone deformity, including grade 4 patellar luxation, to obtain a good radiographic image may be difficult [27]. An anatomic study has demonstrated that the elevation of the distal femur had a significant effect on measured aLDFA at elevations more than 5 degrees as a result of positioning error [26]. Another study regarding femoral head and neck parameters on cadavers reported mean (±SD) positioning errors of 0.93° (±1.92°) in the frontal plane and 2.39° (±1.13°) in the sagittal plane [28].

The femoral neck angle or angle of inclination plays a significant role in transferring biomechanical forces to the acetabulum [29]. Several methods have been suggested for the measurement of this angle, and different mean values have been reported in sound and unhealthy dogs, including dysplastic and non-dysplastic dogs [14, 29–34]. The SYMAX method was devised by Rumph and Hathcock and is based on the symmetric axis-based procedure [14]. Sarierler reported that there is no significant difference between dysplastic and non-dysplastic dogs using the SYMAX method [29]. The authors reported that the SYMAX method was more accurate and measurements were most consistent compared with other methods. In our study, a high intra- and inter-observer correlation was recorded for femoral neck angle with SYMAX method. Percentages of intra-observer group ranges, which were smaller than 4° for the first, second and third observer, were 71.79%, 90%, 95.12%, respectively, and the percentage for inter-observer variations was 92.3%.

The proximal femoral angles in the frontal plane, including aLPFA and mLPFA, could be used to evaluate the shape and deformities of the proximal femur [15]. These angles could also be used to evaluate the bone healing procedure after surgical intervention or fractures of the proximal femur. The greater trochanter of the femur is an important landmark in measurements of proximal femoral alignment. Positioning of the femur can influence the relative position of the greater trochanter on radiographs [15]. Tomlinson et al. [15] reported that there is a significant anatomic difference between the shape of the greater trochanter and femoral head in four large breed dogs. In another study, a significantly higher mLPFA range was recorded for female dogs compared to male dogs [11]. In another study, a significantly decreased aLPFA for Yorkshire terriers with grade 4 MPL was reported [35]. In the present study, high intra- and inter-observer ICC was recorded for aLPFA. The percentage of intra-observer group ranges that were smaller than 4° for the first, second and third observers were 63.15%, 80%, and 87.80%, respectively, and the inter-observer variation was 73.17%. For mLPFA, a good intra-observer ICC for the first observer and high intra-observer ICCs for the second and third observers were recorded; however, the inter-observer ICC was high for this angle. The percentages of intra-observer group ranges that were smaller than 4° for the first, second and third observer were 55.26%, 90%, and 85.36%, respectively, and the inter-observer variability was 61.53%.

According to the literature, the incidence of femoral varus and valgus deformities in the distal part of the femur is greater than in proximal portion, and evaluation of distal femoral angles, including aLDFA, mLDFA, and FVA, are significant [18,36]. Yasukawa et al. [18] reported significantly higher aLDFA, mLDFA, and FVA ranges for Toy poodles with grade 4 MPL compared with sound Toy poodles. The same results have been reported for the same angles in Pomeranians with grade 3 MPL compared to grade 1, 2 and control groups [23]. Žilinčík et al. [35] reported significantly greater values for aLDFA and FVA in Yorkshire terriers with grade 4 MPL compared with other groups. Another study reported that the mean values for aLDFA in small breed dogs with grade 4 MPL were significantly higher than in other groups [37]. The results reported by Phetkaew et al. [38] showed that mLDFA was relevant to the severity of MPL, which admits the results reported for Toy poodles and English bulldogs. Previous studies have shown a relationship between varus deformity of the distal femur and the severity of MPL [1,5,39,40]. Continuous pressure on the distal femoral physis produced by malalignment of the quadriceps muscles and MPL may generate or worsen femoral varus deformities [1, 39]. In the present study, a high inter-observer correlation was recorded for aLDFA, mLDFA and FVA; however, the intra-observer correlation for aLDFA, mLDFA, and FVA were good for the first observer and high for the second and third observers. The aLDFA intra-observer group ranges that were smaller than 4° for the first, second and third observer were 87.37%, 100%, and 97.56%, respectively, and the inter-observer variability was 80.48%. The mLDFA intra-observer group ranges that were smaller than 4° for the first, second and third observer were 87.17%, 100%, and 97.56%, respectively, and the inter-observer variability was 90%. The FVA intra-observer group ranges that were smaller than 4° for the first, second and third observer were 78.94%, 97.5%, and 97.56%, respectively, and the inter-observer variability was 76.92%.

The angle of anteversion (AA) is an important index to assess the torsion of the femur. Femoral torsion may be inward (increased femoral anteversion) or outward (retroversion). Different methods have been reported for measurements of AA, and the radiographic method is the easiest method. However, when compared to other imaging techniques, such as computed tomography and magnetic resonance imaging, which are the gold standards in human medicine [10], radiographs may not be accurate because of difficulties in positioning the femur. Dudley et al. [10] reported that there is no significant difference among different methods, including radiography, CT and anatomic preparation, in healthy dogs. Other studies reported a significantly lower AA range for Yorkshire terriers and Toy poodles with grade 4 MPL [18,35]. In the present study, a high inter-observer correlation was recorded for AA. A good intra-observer correlation for the first observer and high intra-observer correlations for the second and third observers were recorded. Intra-observer group ranges of AA that were smaller than 4° for the first, second and third observer were 50%, 100%, and 60%, respectively, and the inter-observer variation was 68.42%.

The relationship between the incidence of MPL and proximal tibial varus or valgus has not been definitively proved, but proximal tibial valgus is characteristic of dogs with MPL [2,8]. Yasukawa et al. [18] reported no significant difference for mMPTA and mMDTA in Toy poodles with and without MPL, whereas Olimpo et al. [37] reported high mMPTA for small breed dogs with grade 4 MPL. Lambert and Wendelburg [41] reported that identification of varus deformities in the proximal tibia using mMPTA with a tangential caudocranial projection was possible, whereas the deformities were not identified in a straight caudocranial projection. However, it has been reported that internal and external rotation of the tibia cause underrated and overrated mMPTA, respectively [41].

Alignments of the tibia in the sagittal plane, such as mCrPTA, mCdPTA, mCrDTA and mCdDTA, are used to indicate procurvatum and recurvatum of the tibia [42]. Previous studies described an existence of caudal deformity of the proximal tibia in small breed dogs, which increases the risk of the cranial cruciate ligament rupture [43]. In another study, a higher mCdPTA was reported for small breed dogs affected with MPL, but it was not clear if there was a significant relationship between caudal tibial deformity and the incidence of the MPL [37].

In the literature, a higher DPA was reported for sound small breed dogs compared with dogs with cranial cruciate ligament rupture and normal large breed dogs. Evaluation of the DPA may illustrate the proximal shaft deformity of the tibia [44].

In the present study, a high inter-observer correlation was recorded for mMPTA, mMDTA, mCrPTA, and mCdPTA. For the other angles, including mCrDTA, mCdDTA and DPA, good correlation was recorded. This shows that measurement of the proximal tibia may be more accurate than measurement of the distal tibia. Intra-observer correlations for tibial alignment in our study for the first observer were 14.28% high correlations and 57.14% good correlations. 100% of the measurements of the second observer had a high correlation, and for the third observer, 42% of correlations were high and 28% of correlations were good.

The authors had different study limitations in this study such as the limited number of the radiographs and absence of the control group. All dogs used in this study had a bilateral MPL. Of course, it would be of interest to also obtain data from unaffected limbs; however, no radiographs of healthy dogs were available in the patient material. Unfortunately, in the few cases where unilateral MPL was clinically diagnosed, no radiographs of the non-affected limb were taken. This problem arose from the fact that this study is a retrospective study. However, this should be addressed in further prospective studies.

The low sample size is justified by including only one breed, where every single individual in this study (ICC) is similar to each other. In a consequence the ability to generalize the results to other breeds is limited. In fact, the low sample size is not a limitation at all in this study since the study focusses on the agreement among the observers, which is independent of the data source. The result of the study is statically clear, thus increasing the sample size will have a low influence on the final results. Small sample size can be justified by the selection of the statistical characteristic, for example, if Kappa is used in the study, the number of cases would have to be increased because kappa is based on frequencies. It can be assumed that the main conclusion of the study (intra- and inter-observer agreement) is not influenced by increasing sample size.

The other limitation is the unequal intervals between measurements. The interval for all the observers between the measurement were minimum of two weeks and maximum of 12 weeks between the sessions. Different intervals may be affecting the accuracy of the measurements. Effect of the muscle contracture in dogs with severe grades of MPL is another point, which was discussed before.

## Conclusions

As expressed in this study evaluation of the measurements within and between observers showed that the radiographic method has good to high intra- and inter-observer variability.

## Supporting information

S1 File Intra- and inter-observer group ranges. SYMAX: angle of inclination with SYMAX method, aLPFA: anatomic lateral proximal femoral angle, aLDFA: anatomic lateral distal femoral angle, FVA: femoral varus angle, mLPFA: mechanical lateral proximal femoral angle, mLDFA: mechanical lateral distal femoral angle, AA: angle of anteversion, mMPTA: mechanical medial proximal tibial angle, mMDTA: mechanical medial distal tibial angle, mCrPTA: mechanical cranial proximal tibial angle, mCdPTA: mechanical caudal proximal tibial angle, mCrDTA: mechanical cranial distal tibial angle, mCdDTA: mechanical caudal distal tibial angle, DPA: distal tibial axis/proximal tibial axis angle.
(PDF)
