# Supplementary material for: Femoral and tibial alignments in chihuahuas with patellar luxation by radiograph: Angular values and intra- and inter-observer agreement of measurements
Source: PLoS One. 2019 Mar 28;14(3):e0214579. doi: 10.1371/journal.pone.0214579 (PMC6438527; doi:10.1371/journal.pone.0214579)

Intra-observer group ranges

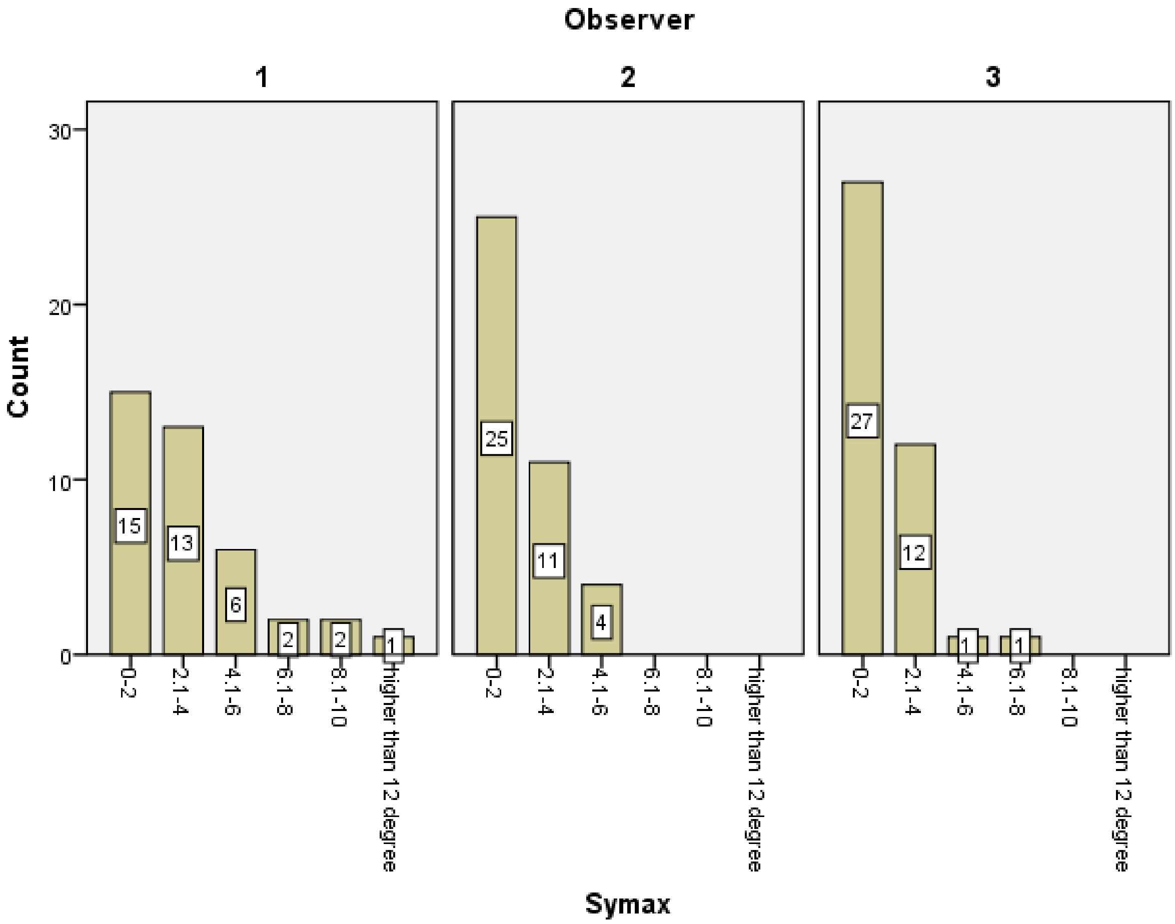

Inter-observer group ranges

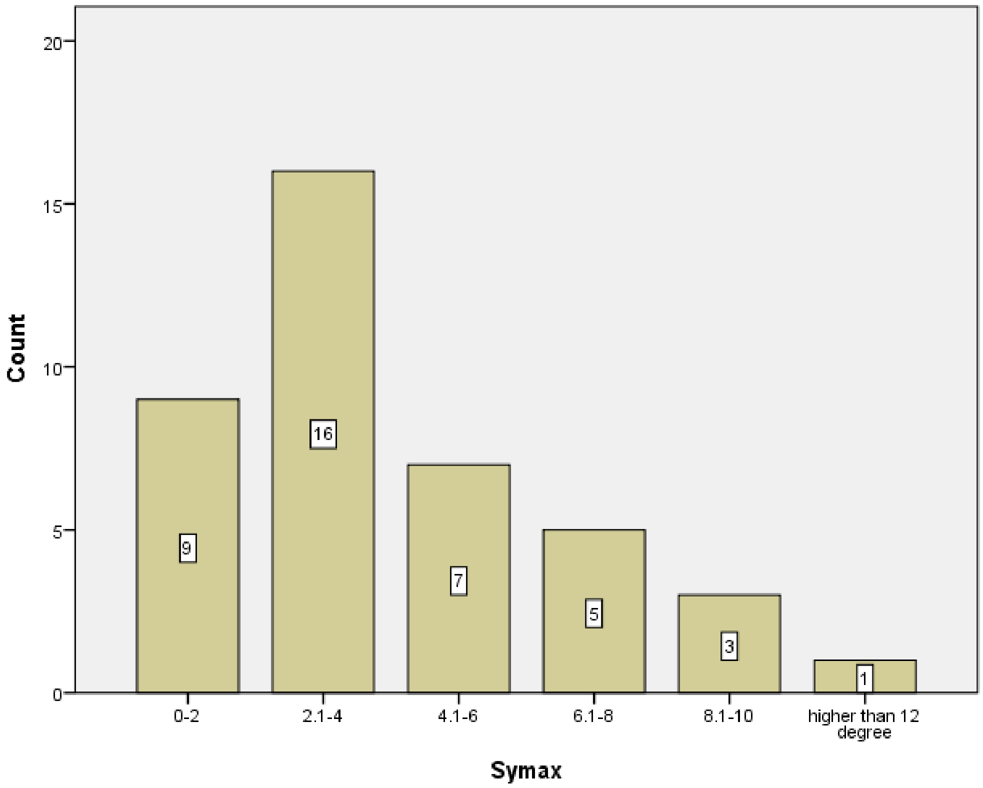

Intra-observer group ranges

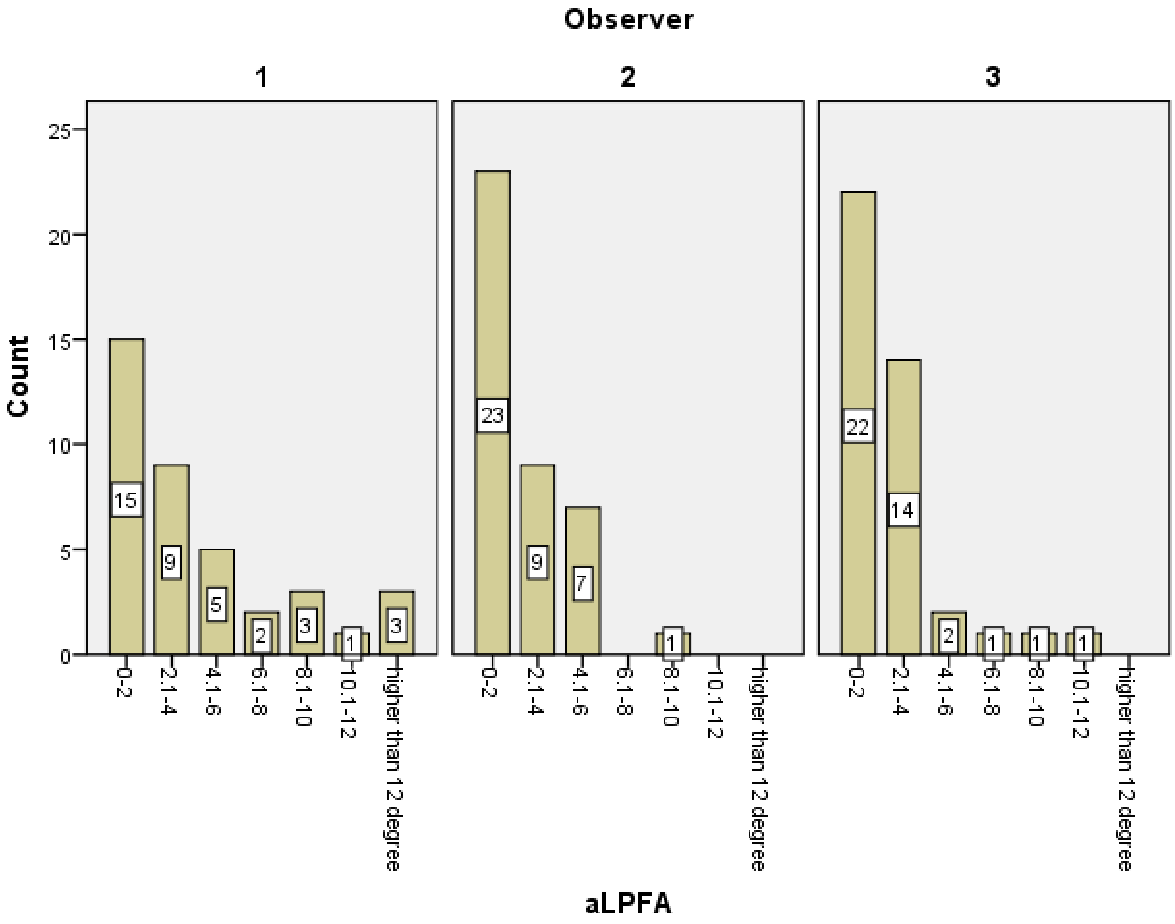

Inter-observer group ranges

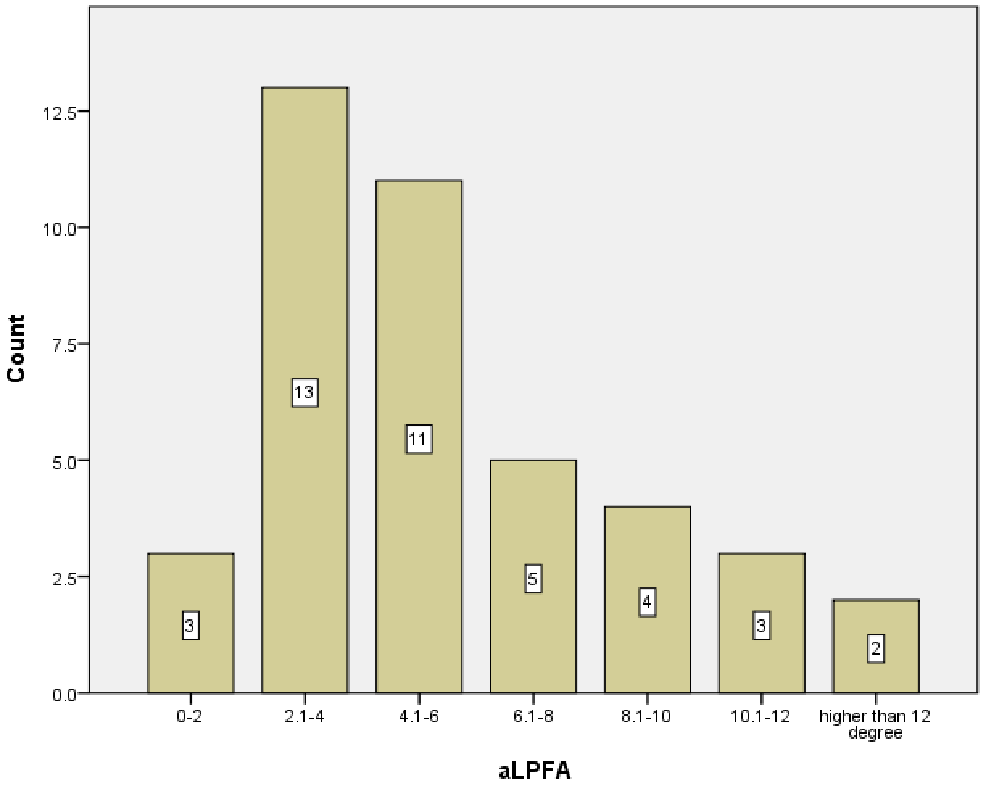

Intra-observer group ranges

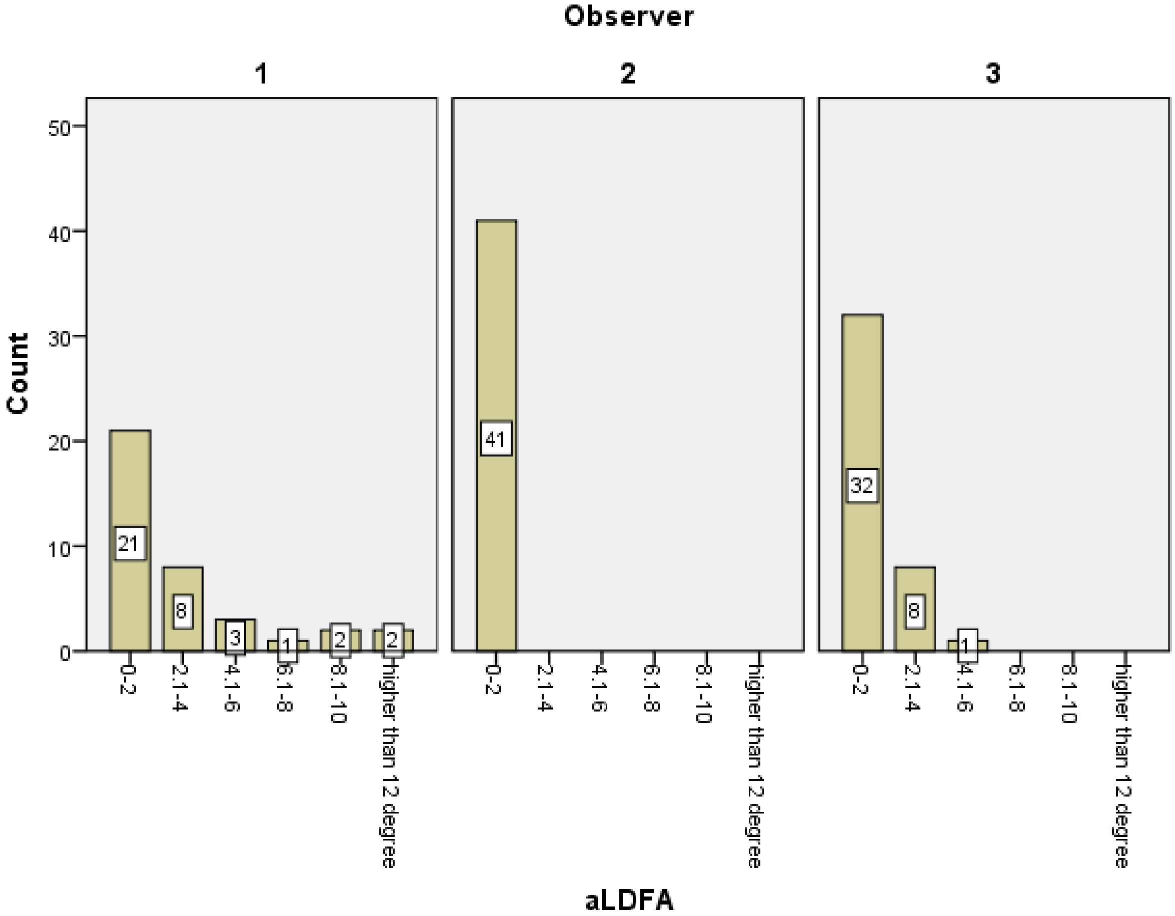

Inter-observer group ranges

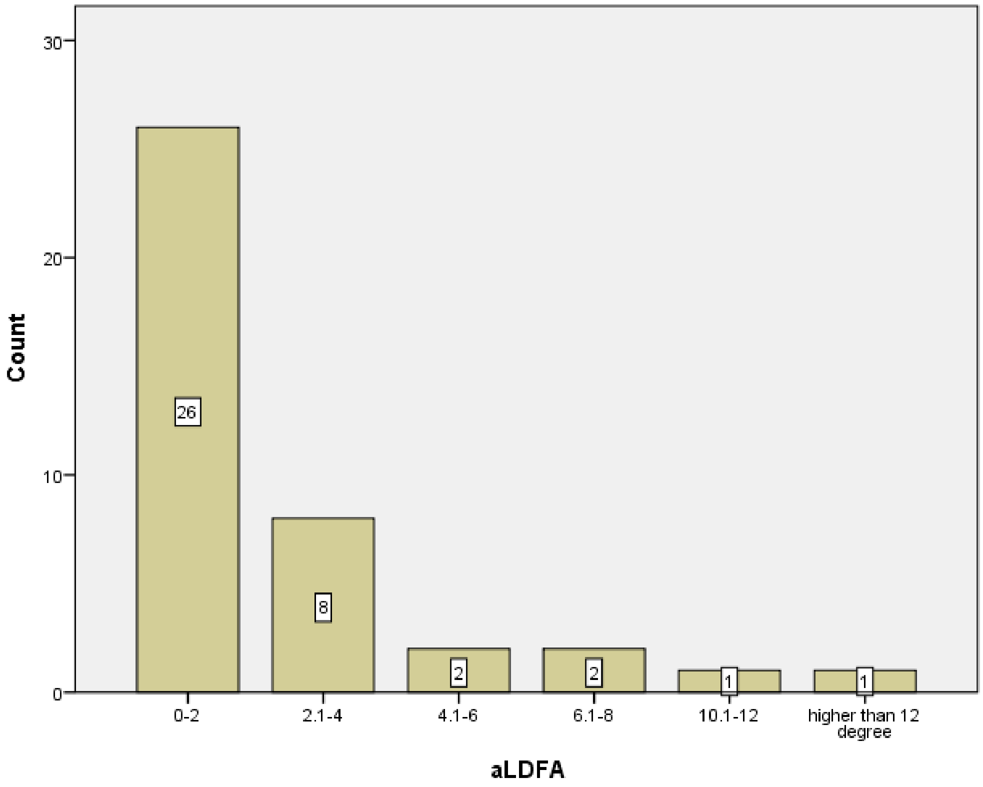

Intra-observer group ranges

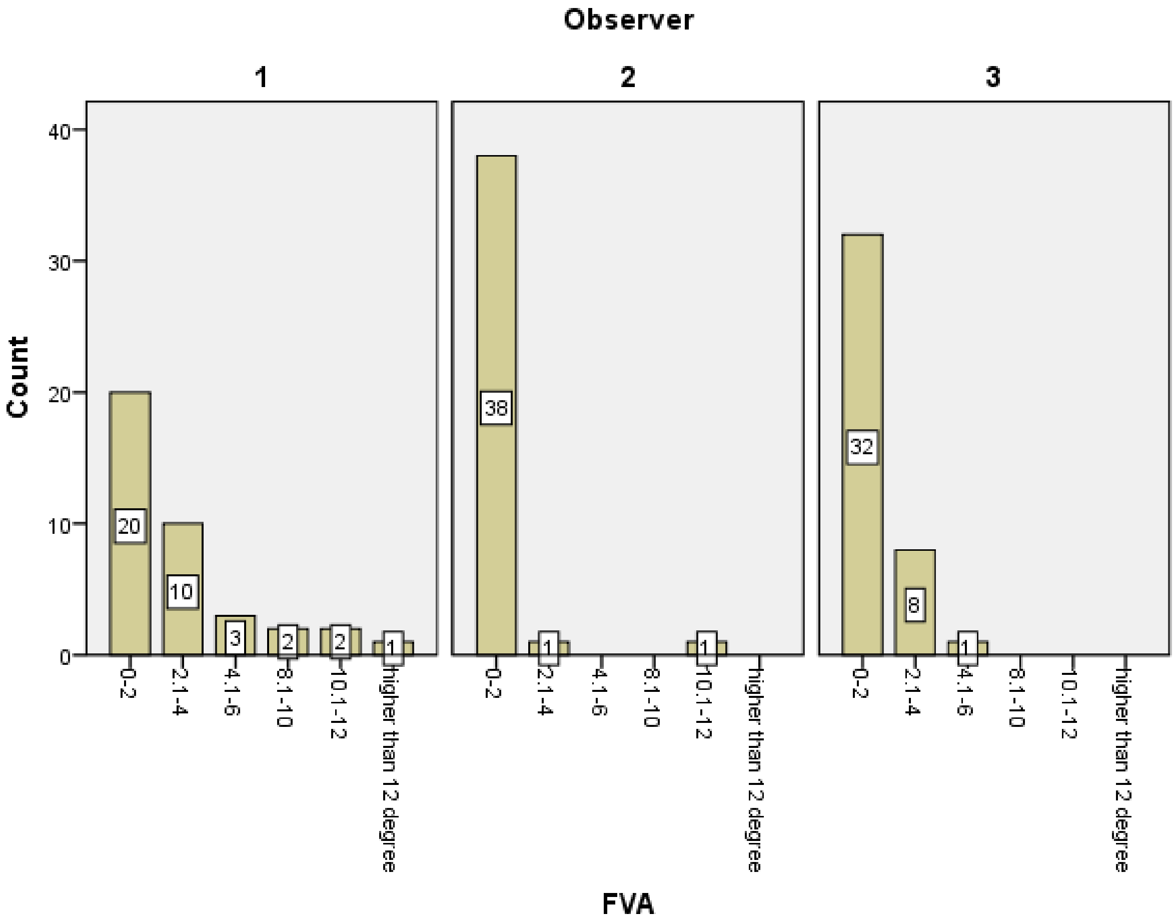

Inter-observer group ranges

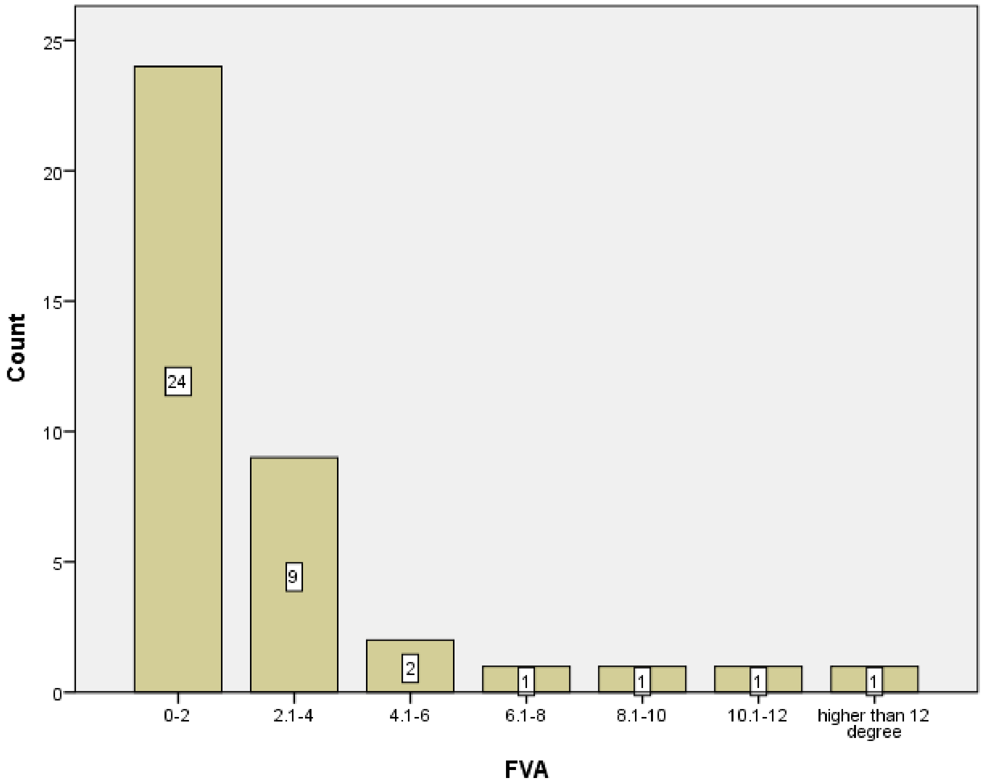

Intra-observer group ranges

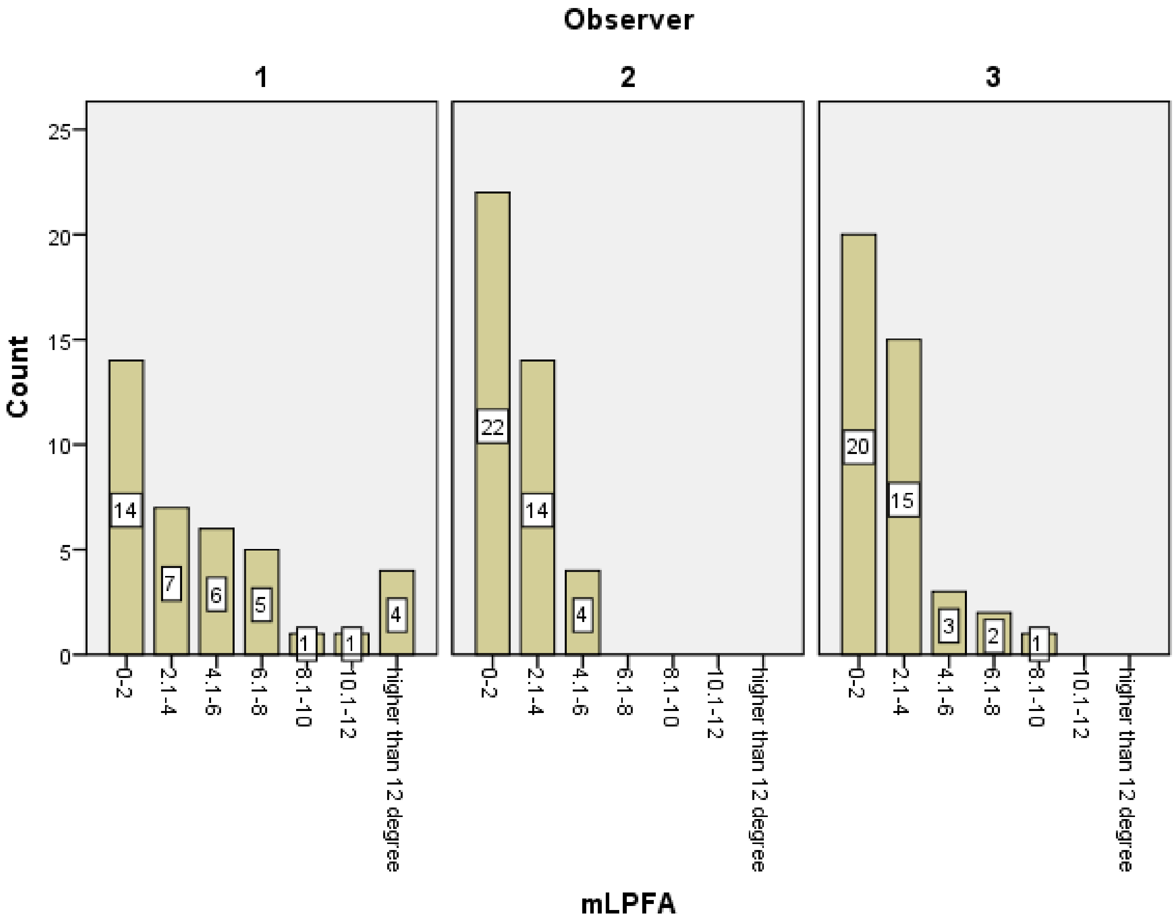

Inter-observer group ranges

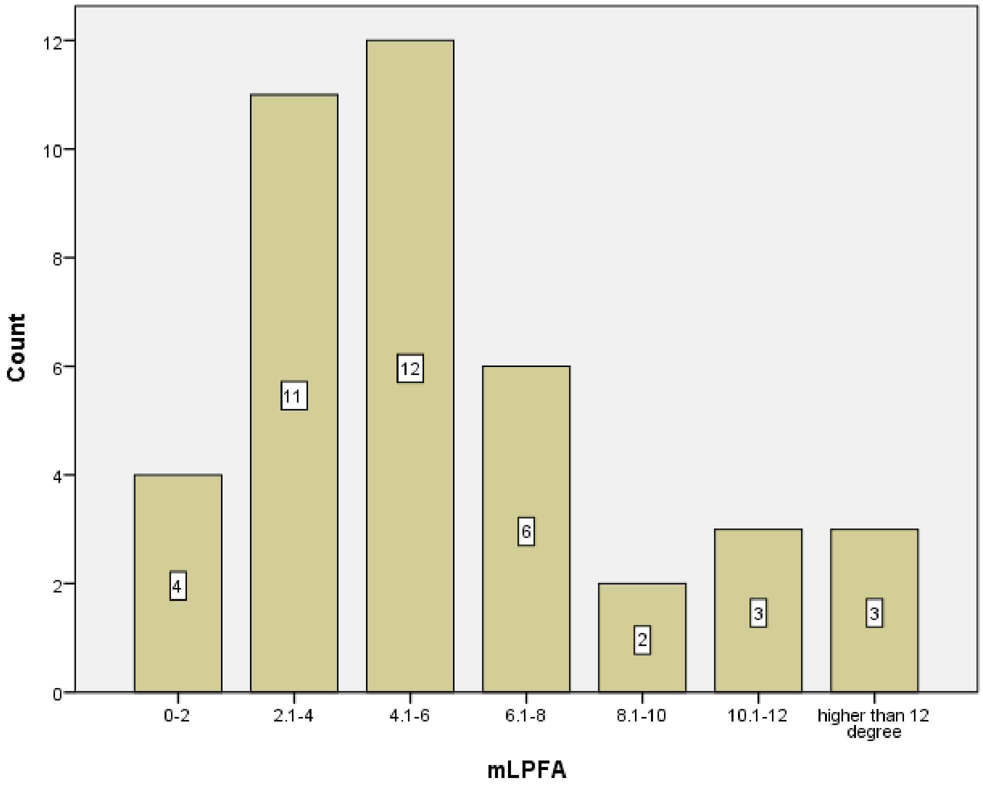

Intra-observer group ranges

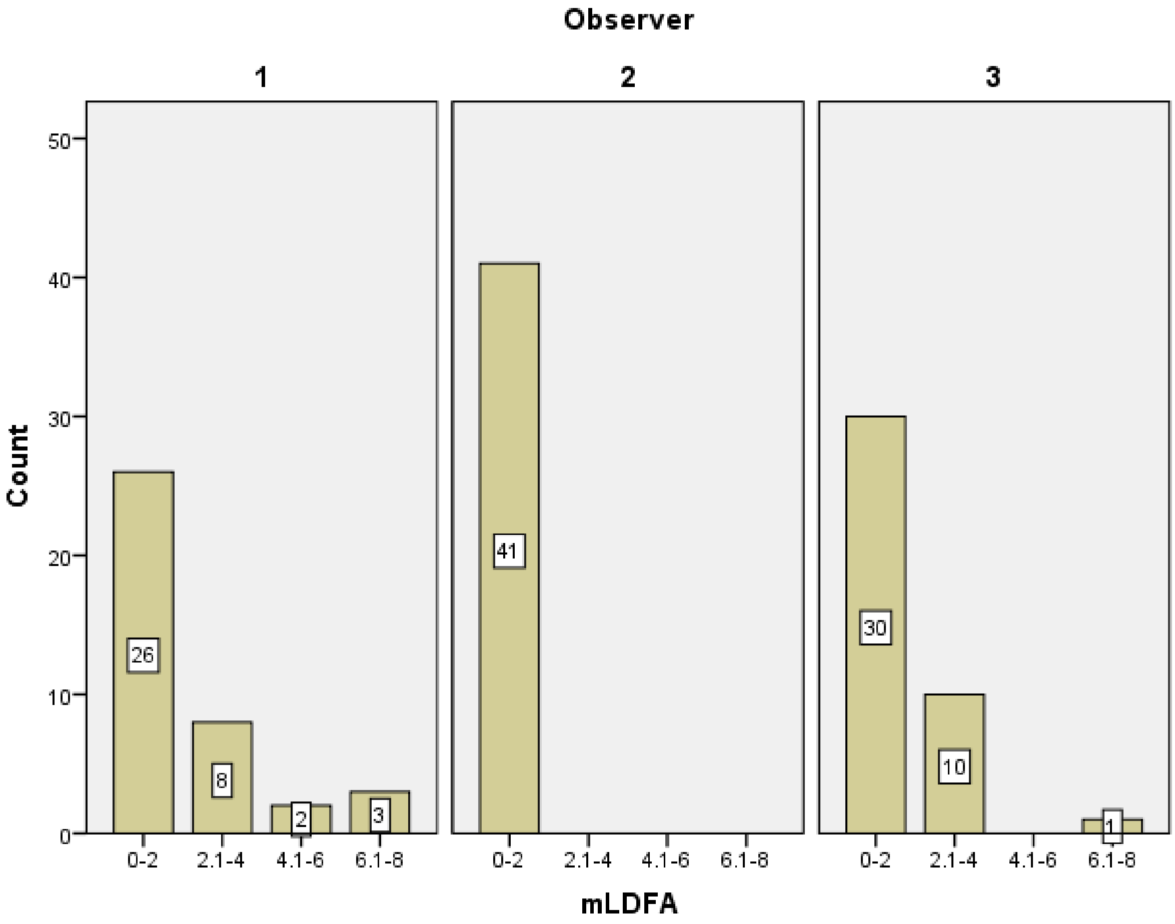

Inter-observer group ranges

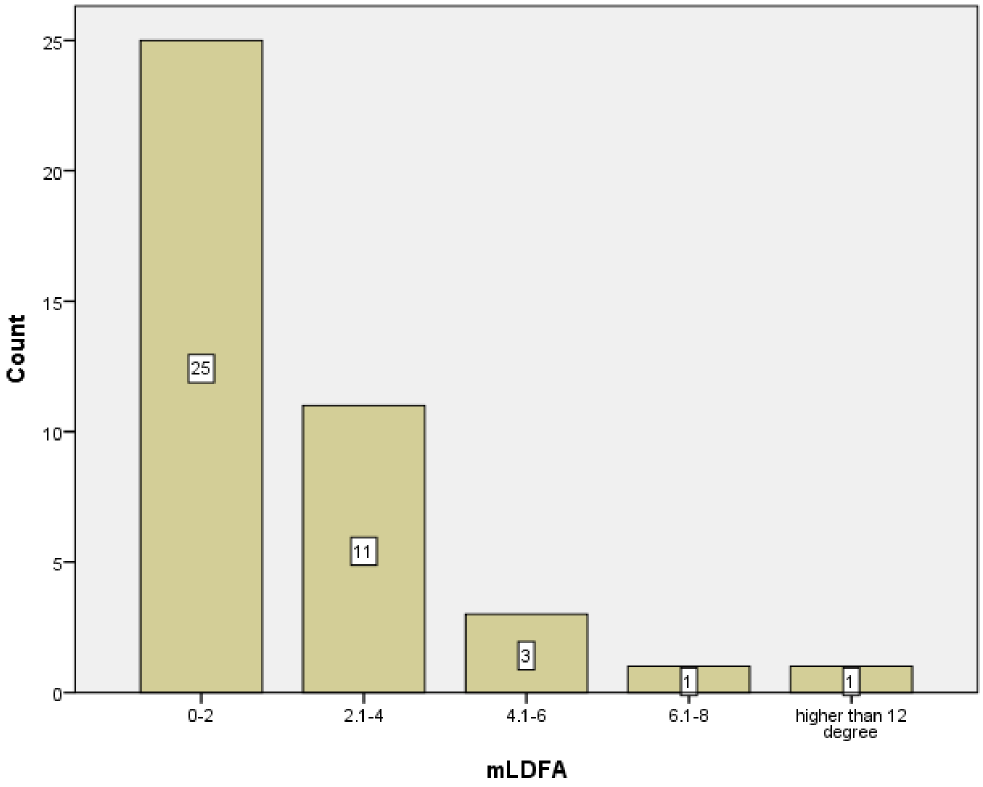

Intra-observer group ranges

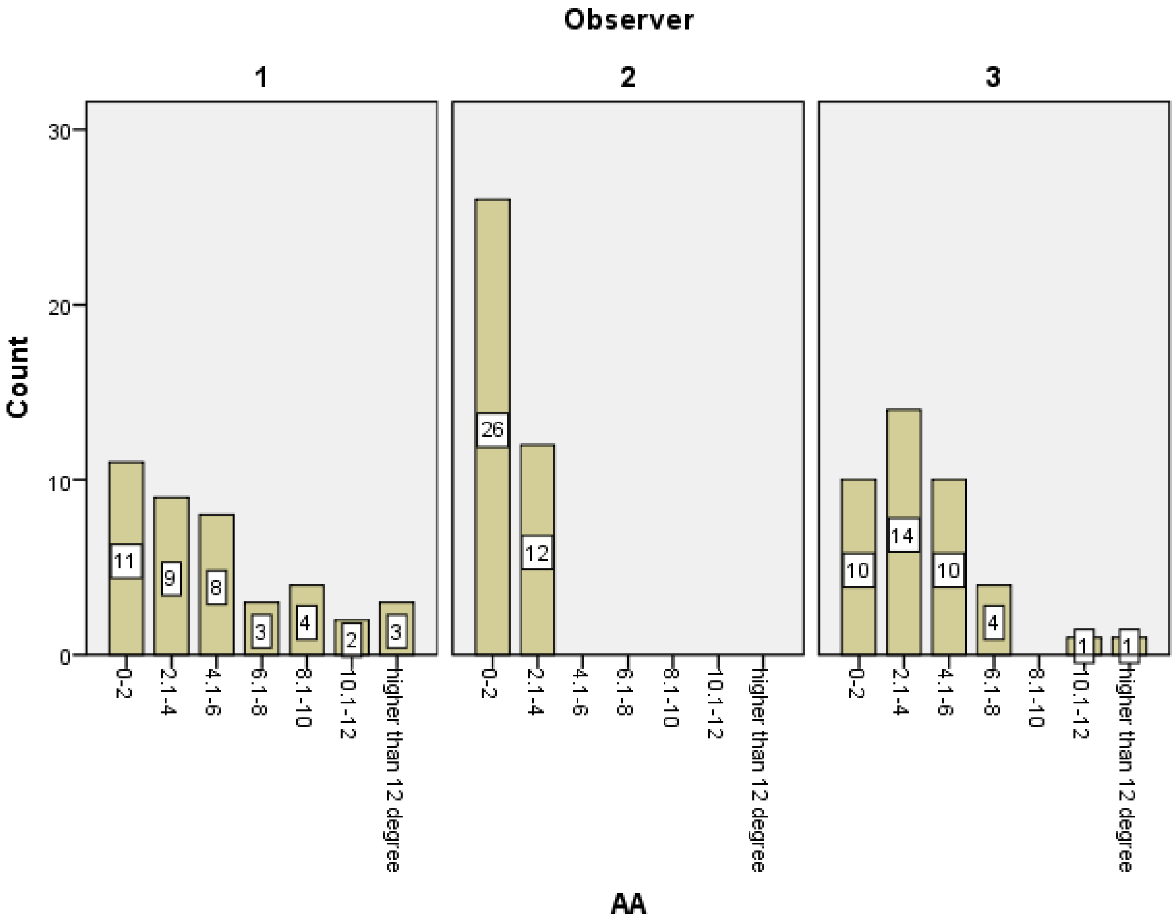

Inter-observer group ranges

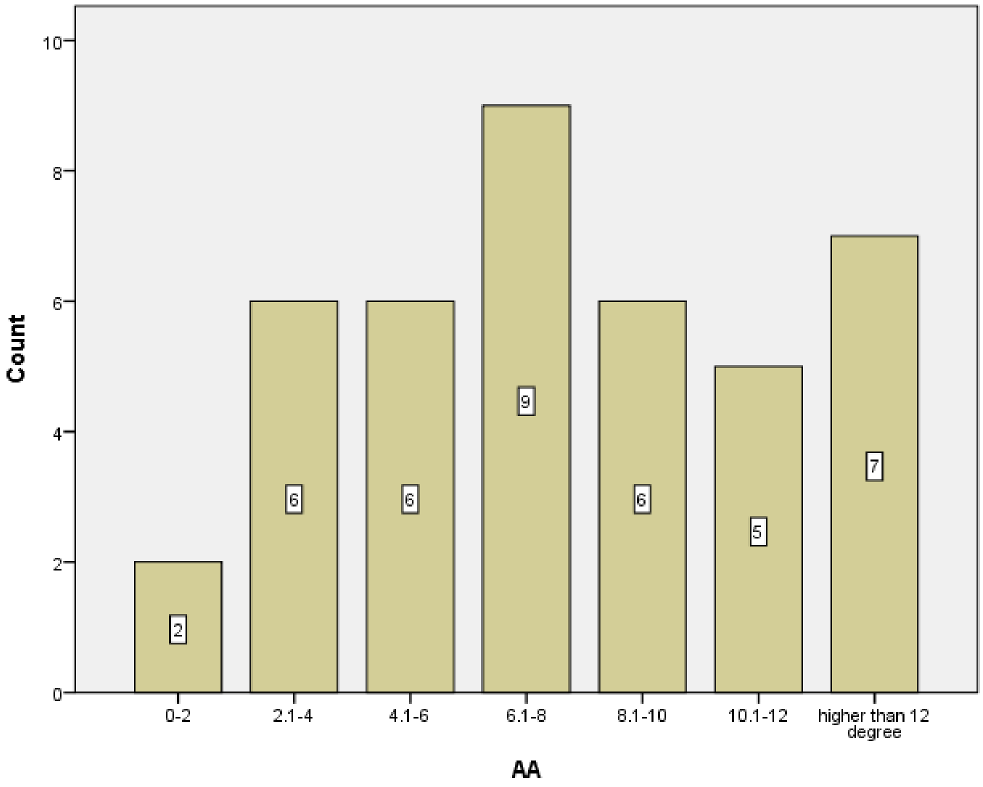

Intra-observer group ranges

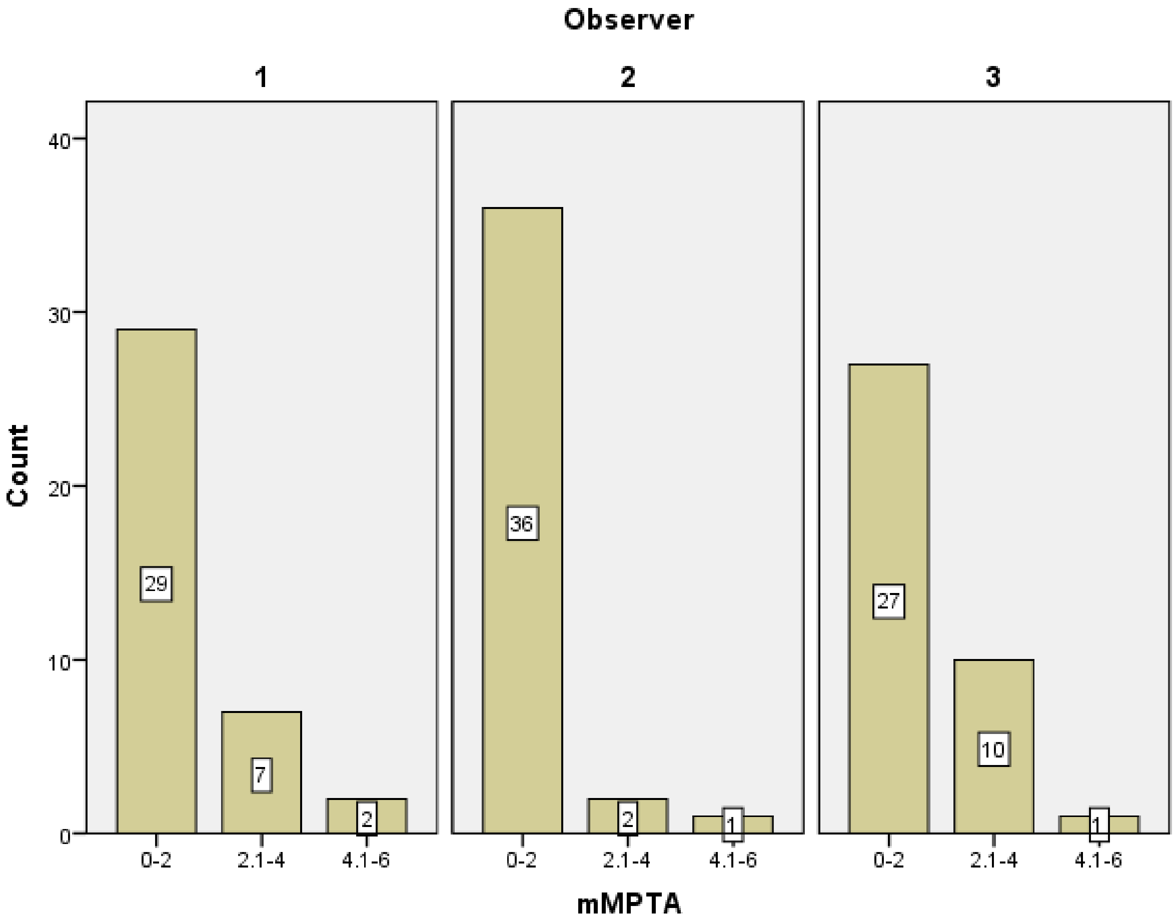

Inter-observer group ranges

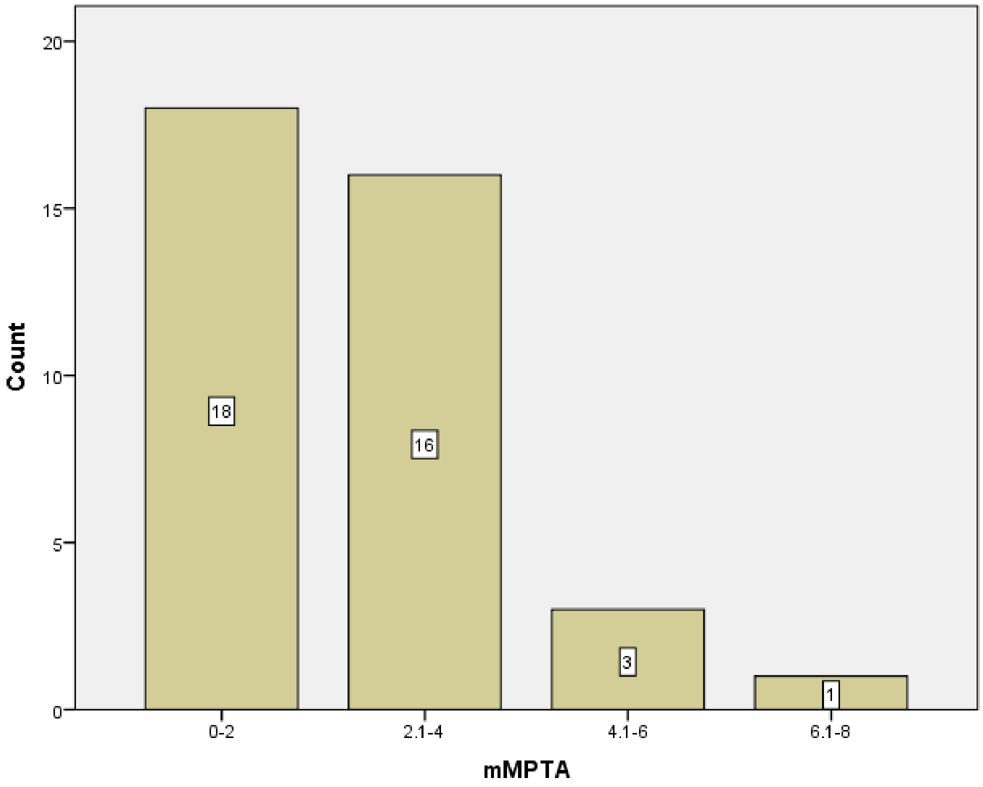

Intra-observer group ranges

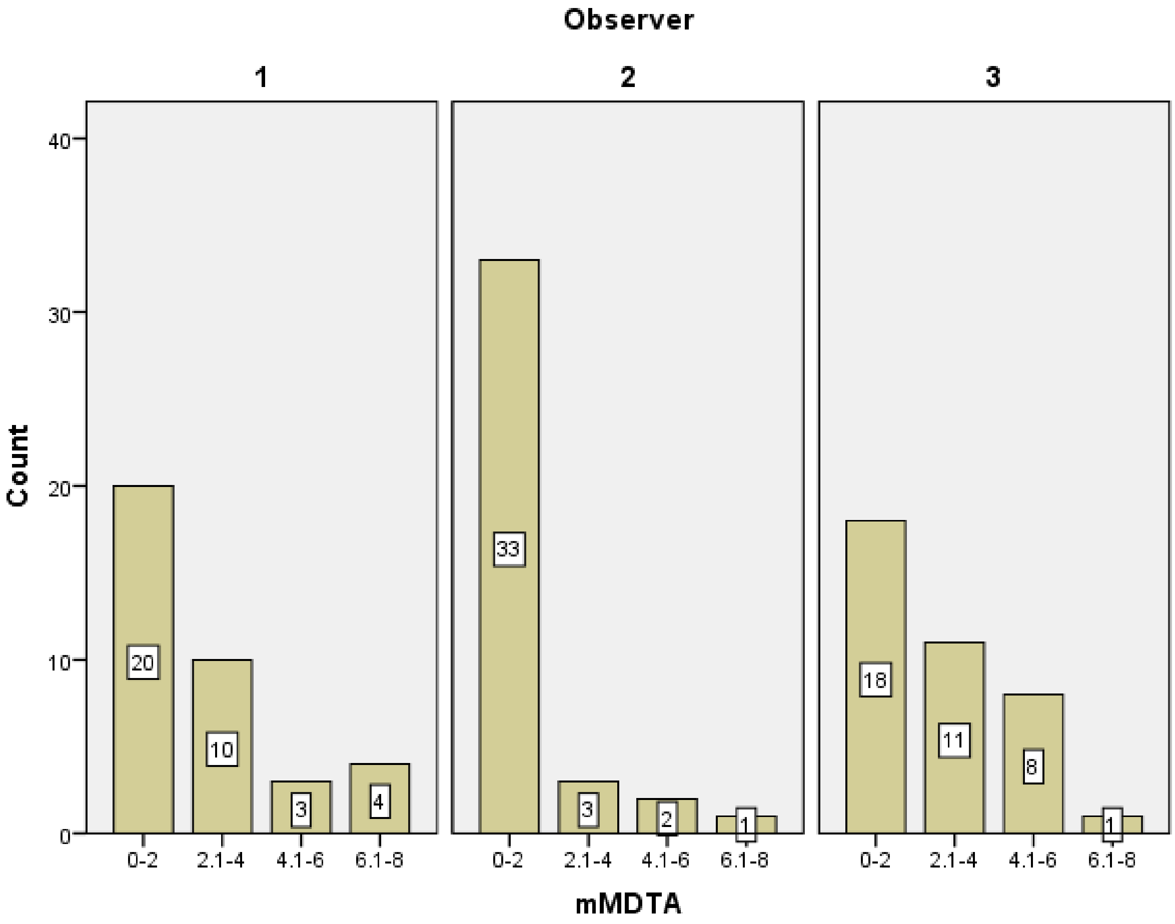

Inter-observer group ranges

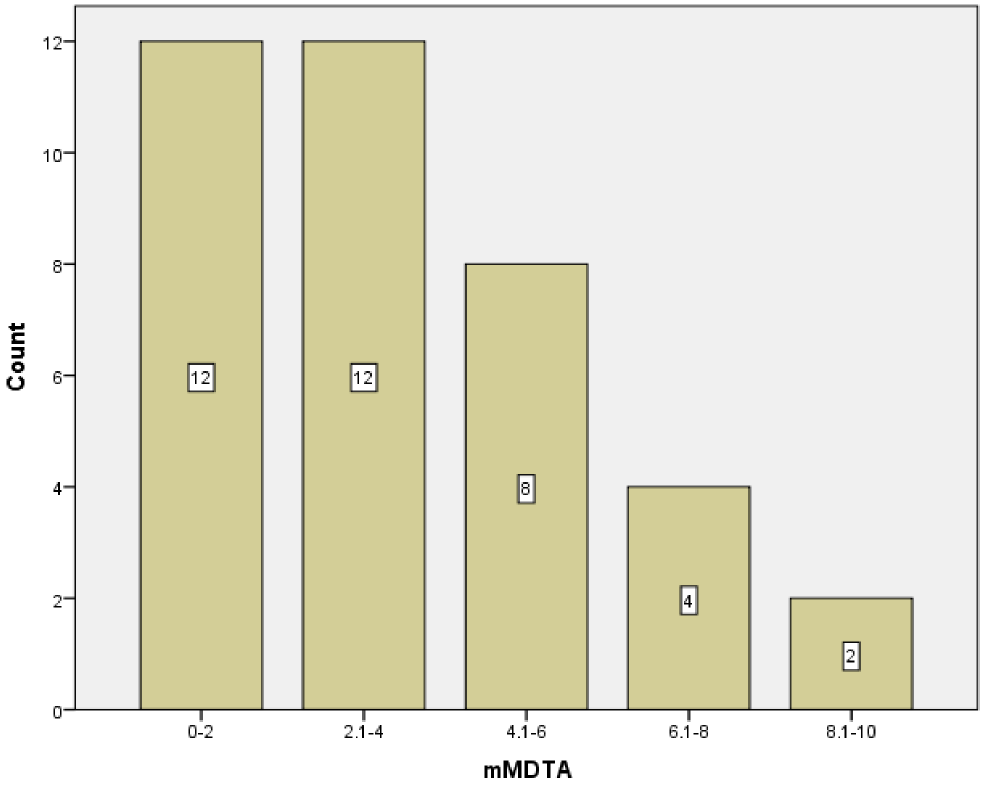

Intra-observer group ranges

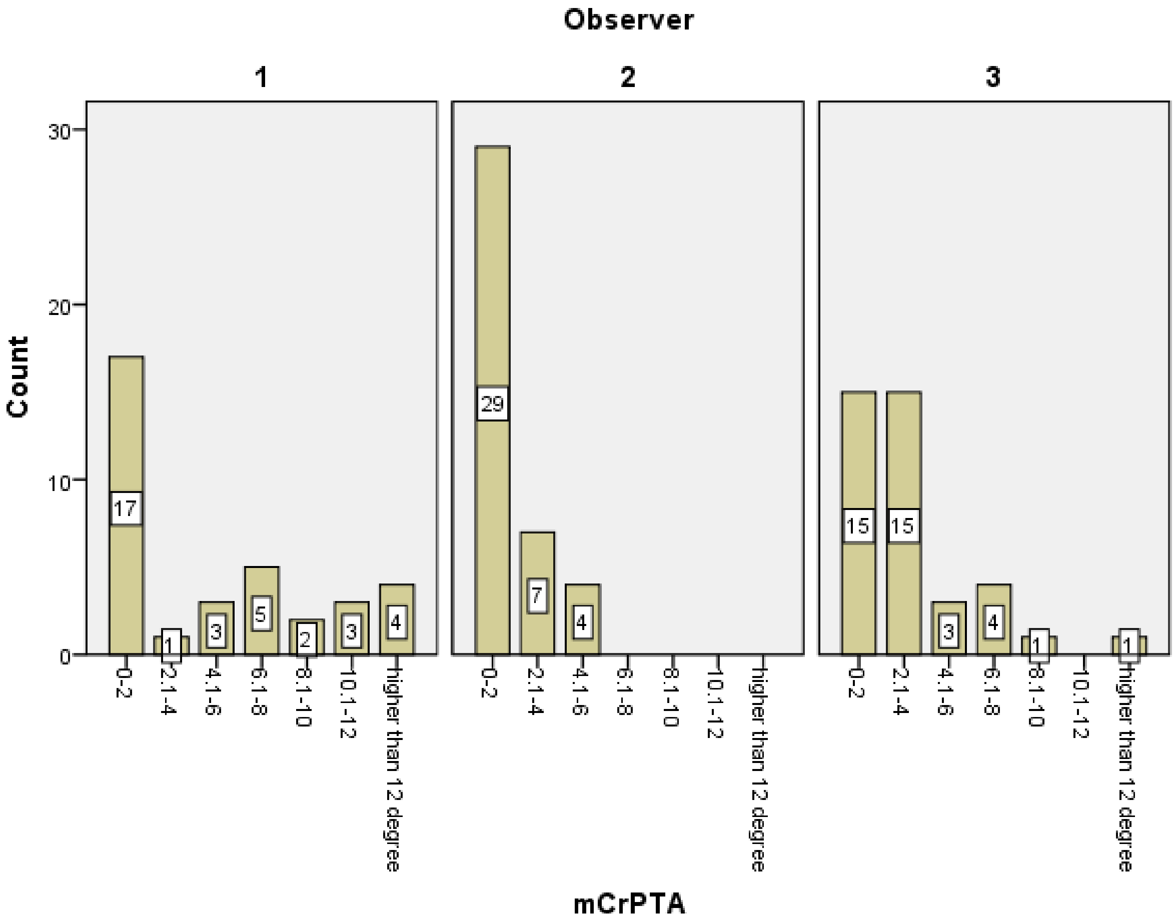

Inter-observer group ranges

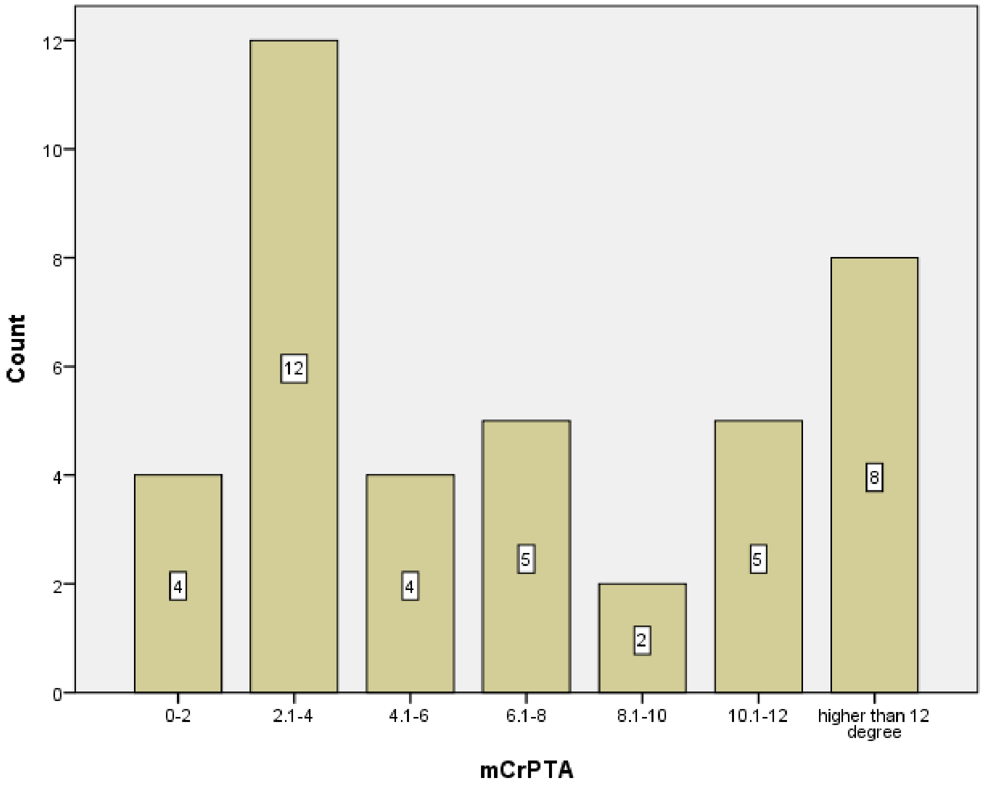

Intra-observer group ranges

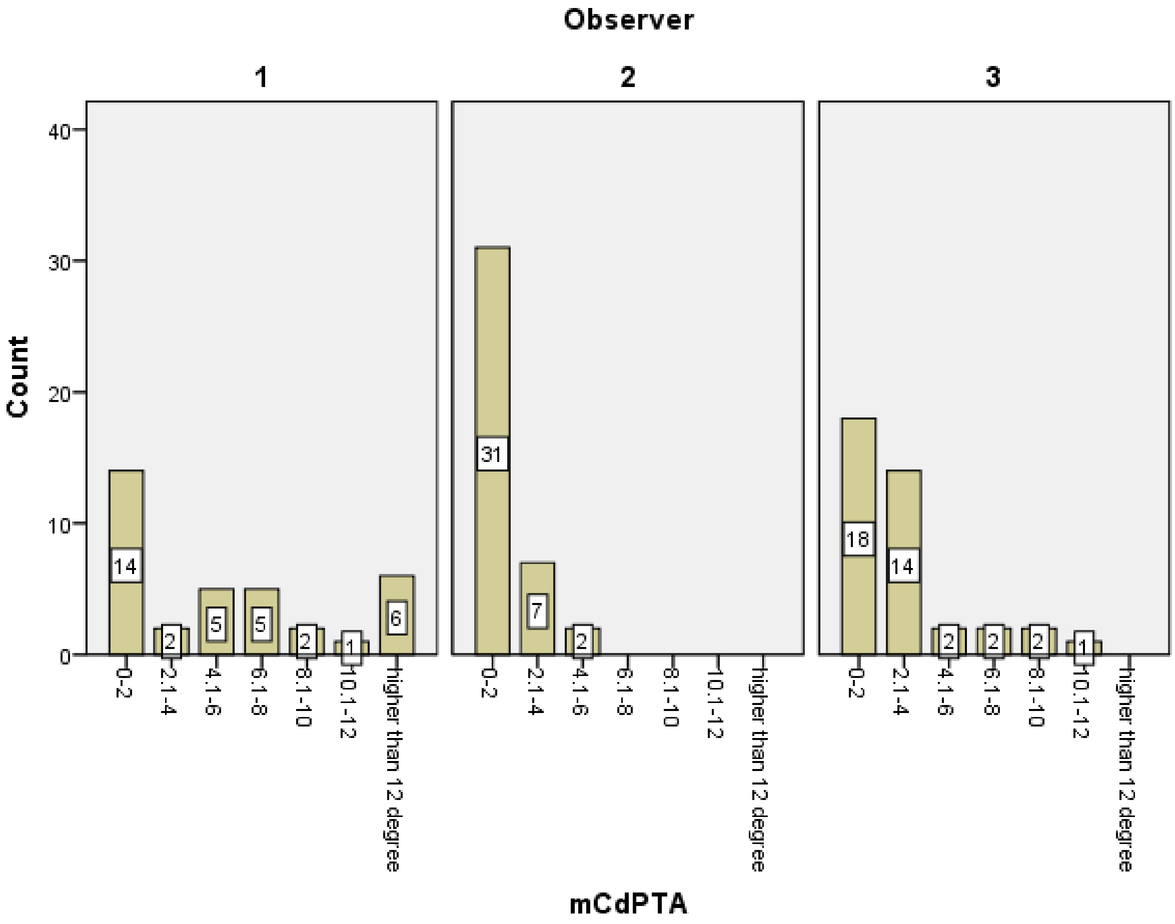

Inter-observer group ranges

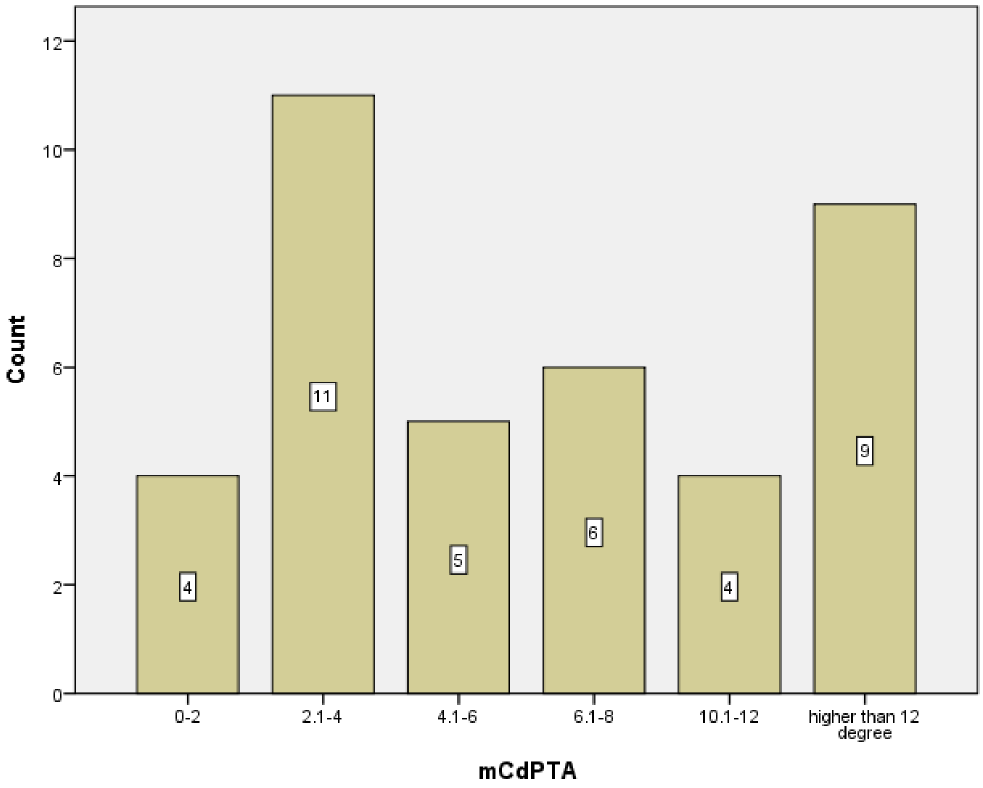

Intra-observer group ranges

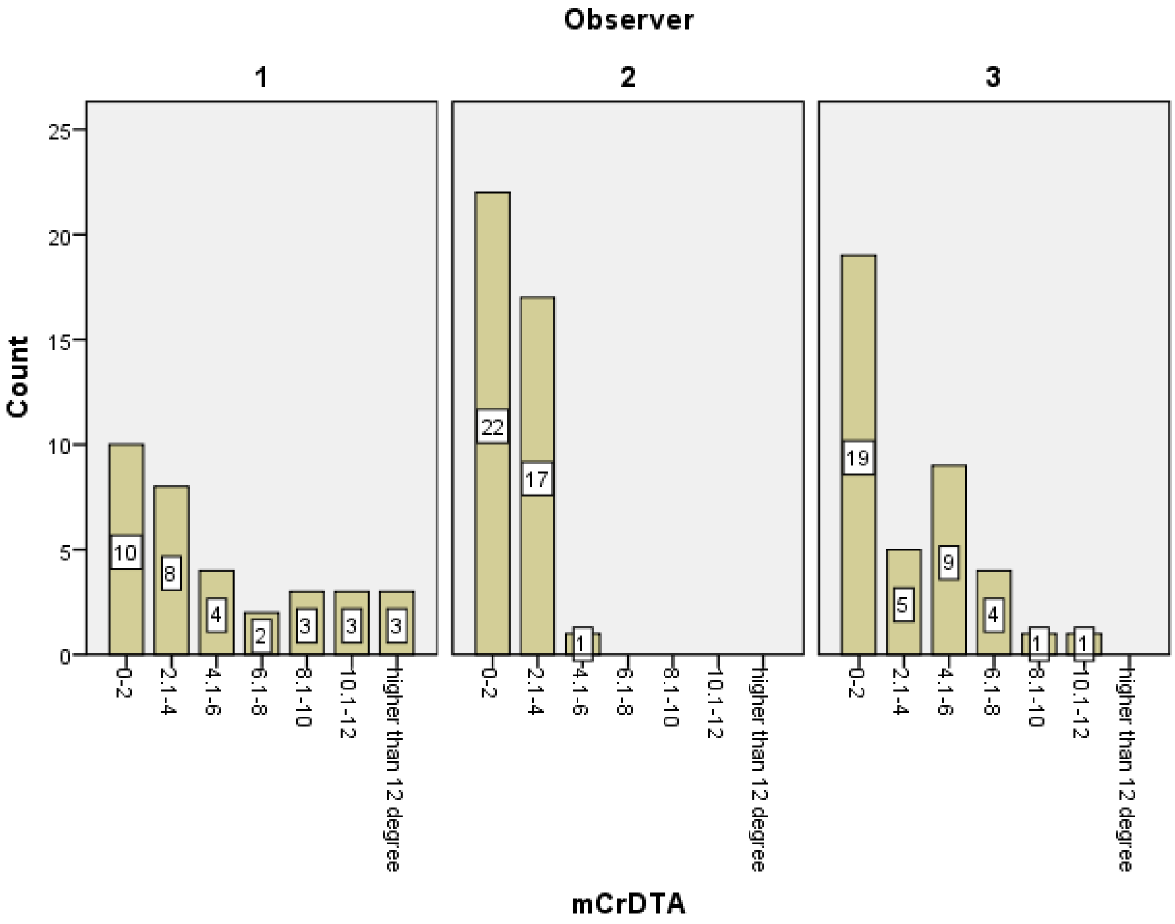

Inter-observer group ranges

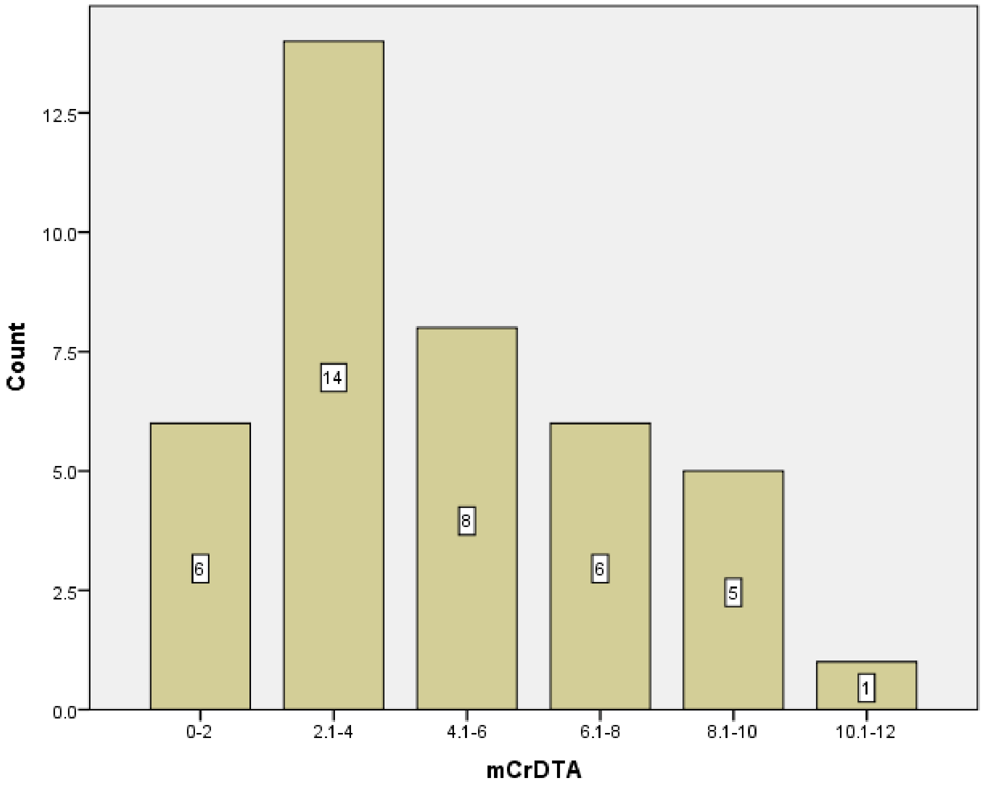

Intra-observer group ranges

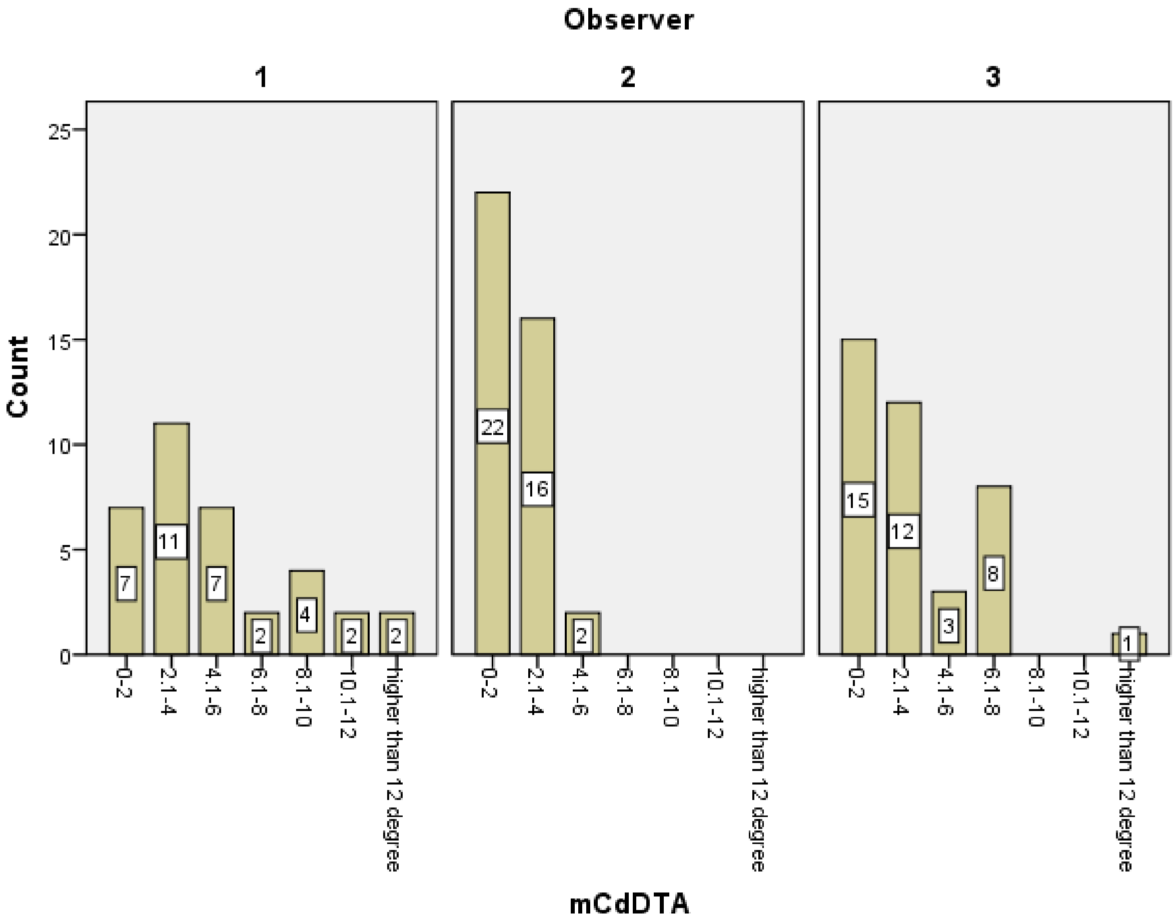

Inter-observer group ranges

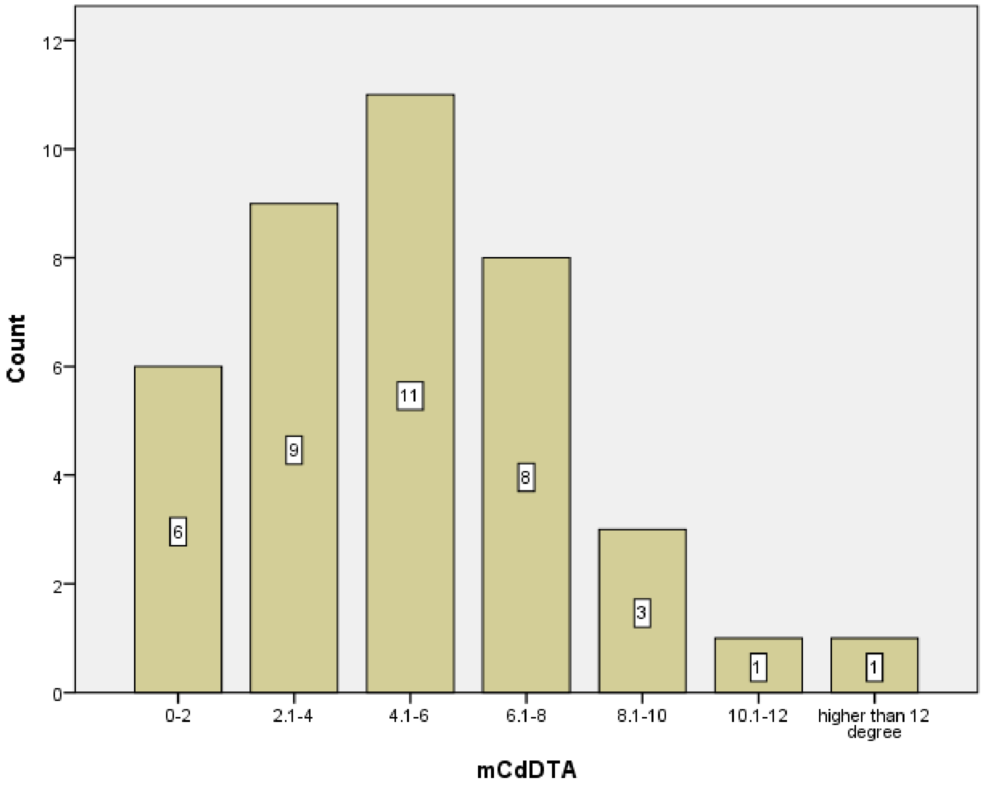

Intra-observer group ranges

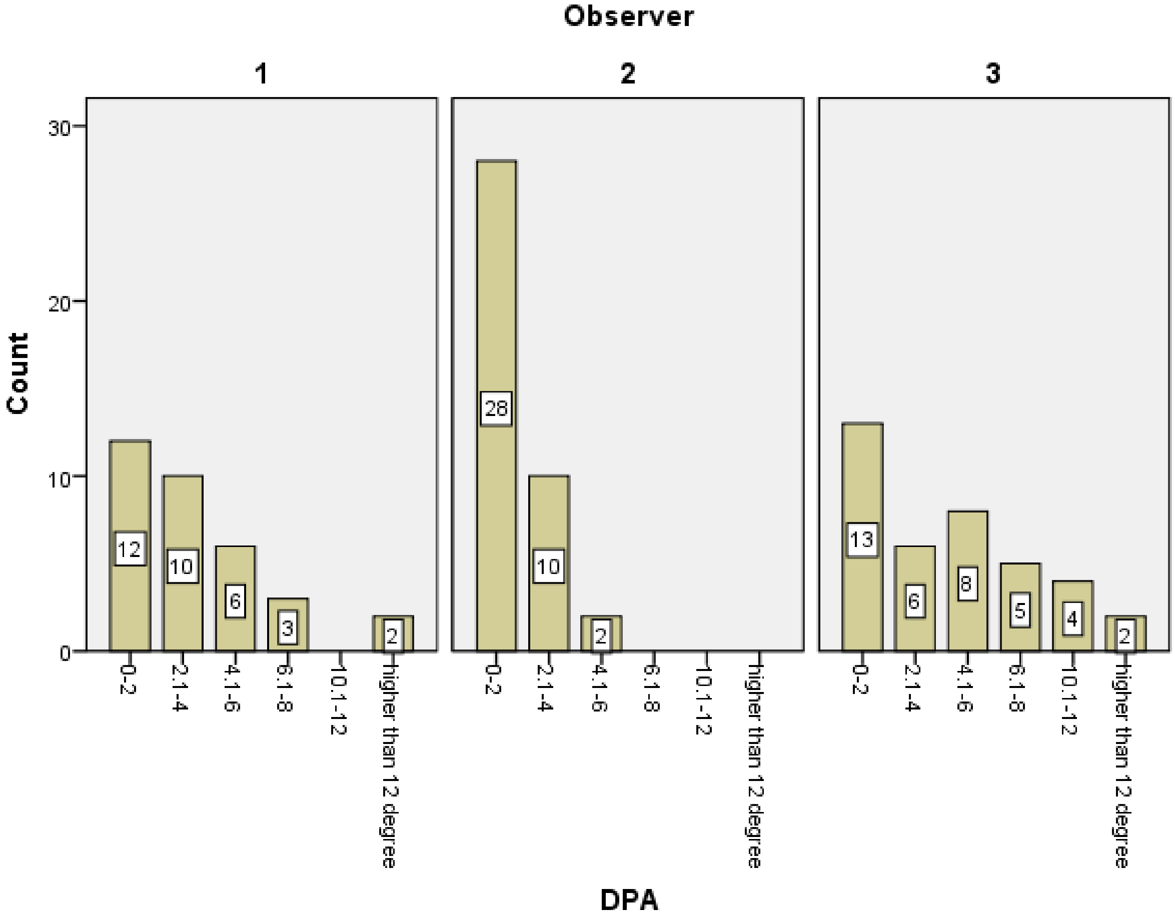

Inter-observer group ranges

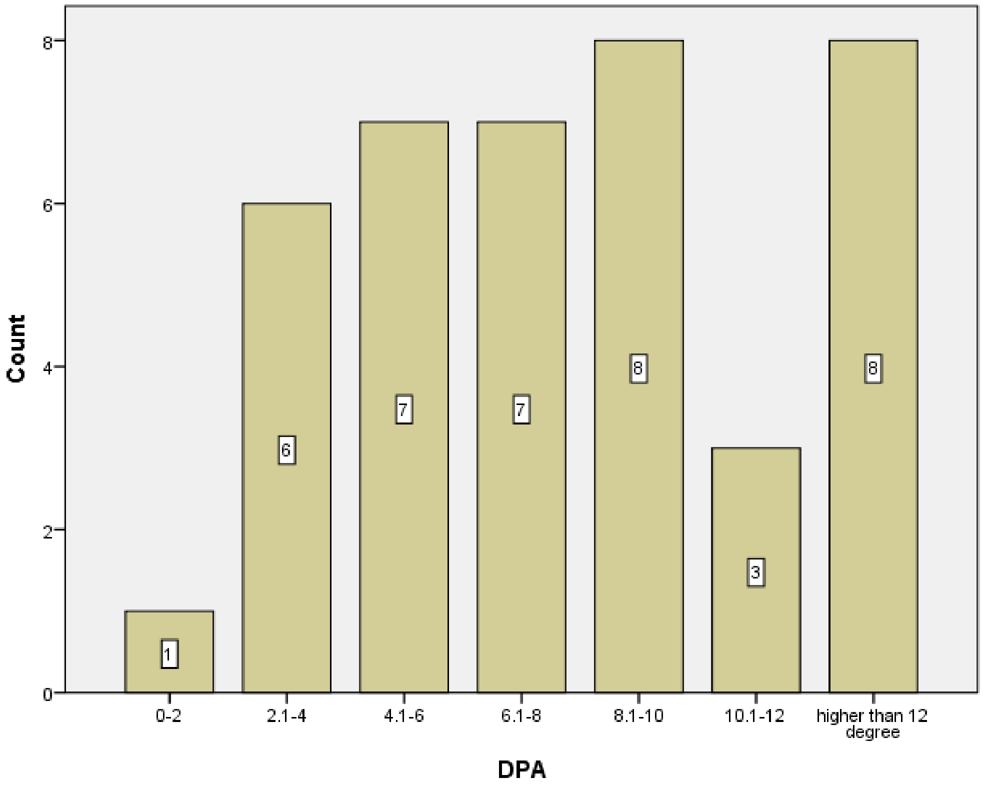

Supplement: S1 File — SYMAX: angle of inclination with SYMAX method, aLPFA: anatomic lateral proximal femoral angle, aLDFA: anatomic lateral distal femoral angle, FVA: femoral varus angle, mLPFA: mechanical lateral proximal femoral angle, mLDFA: mechanical lateral distal femoral angle, AA: angle of anteversion, mMPTA: mechanical medial proximal tibial angle, mMDTA: mechanical medial distal tibial angle, mCrPTA: mechanical cranial proximal tibial angle, mCdPTA: mechanical caudal proximal tibial angle, mCrDTA: mechanical cranial distal tibial angle, mCdDTA: mechanical caudal distal tibial angle, DPA: distal tibial axis/proximal tibial axis angle. (PDF) [file pone.0214579.s001.pdf]
